# Testing the adolescent social reorientation model during self and other evaluation using hierarchical growth curve modeling with parcellated fMRI data

**DOI:** 10.1016/j.dcn.2022.101089

**Published:** 2022-02-23

**Authors:** Danielle Cosme, John C. Flournoy, Jordan L. Livingston, Matthew D. Lieberman, Mirella Dapretto, Jennifer H. Pfeifer

**Affiliations:** a Annenberg School for Communication, University of Pennsylvania, United States; b Department of Psychology, University of Oregon, United States; c Department of Psychology, Harvard University, United States; d Department of Psychology, University of Toronto, Canada; e Department of Psychology, University of California, Los Angeles, United States; f Ahmanson-Lovelace Brain Mapping Center, University of California, Los Angeles, United States; g Department of Psychiatry and Biobehavioral Sciences, University of California, Los Angeles, United States

**Keywords:** Adolescence, Social reorientation, Social status, Self-perception, FMRI, Hierarchical growth curve modeling

## Abstract

Adolescence is characterized as a period when relationships and experiences shift toward peers. The social reorientation model of adolescence posits this shift is driven by neurobiological changes that increase the salience of social information related to peer integration and acceptance. Although influential, this model has rarely been subjected to tests that could falsify it, or studied in longitudinal samples assessing within-person development. We focused on two phenomena that are highly salient and dynamic during adolescence—social status and self-perception—and examined longitudinal changes in neural responses during a self/other evaluation task. We expected status-related social information to uniquely increase across adolescence in social brain regions. Despite using hierarchical growth curve modeling with parcellated whole-brain data to increase power to detect developmental effects, we didn’t find evidence in support of this hypothesis. Social brain regions showed increased responsivity across adolescence, but this trajectory was not unique to status-related information. Additionally, brain regions associated with self-focused cognition showed heightened responses during self-evaluation in the transition to mid-adolescence, especially for status-related information. These results qualify existing models of adolescent social reorientation and highlight the multifaceted changes in self and social development that could be leveraged in novel ways to support adolescent health and well-being.

## 1 Introduction

A common characterization of adolescence favored by researchers, parents, and the general public alike is that it is a time during which adolescents’ social worlds shift in emphasis, away from an early focus on primary caregivers and family members, and strongly towards peer relationships and experiences. One impactful formalization of this view is the “social reorientation” model (Nelson et al., 2005), which proposes that both internal (e.g., hormonal) and external (e.g., sociocultural) forces impact neural processes to alter adolescent behavior in this fashion. Social information is expected to be highly salient across the lifespan in social species (and not only of unique importance during adolescence; Nelson et al., 2016), but there should be particular depth and intensity of processing for stimuli that are developmentally relevant in the social environment. This heuristic model has been incredibly generative and has inspired a vigorous program of research examining peer-related developmental changes in, for example, social cognition (Burnett et al., 2011), face processing (Scherf et al., 2012), reward processing (Richards et al., 2013), and social evaluation (Somerville, 2013). At this point, there is substantial evidence *verifying* the plausibility of the social reorientation model across a variety of processes and therefore we believe the field is primed to test this model in ways that have the potential to *falsify* its central propositions. In particular, this model proposes that social stimuli related to peer integration and acceptance specifically should become uniquely salient during early adolescence (Nelson et al., 2016) compared to other stimuli and other developmental windows (e.g., childhood or late adolescence). Although it can be challenging to derive specific predictions from heuristic models that have the potential to be falsified (van den Bos and Eppinger, 2016), attempting to do so is critical not only for model refinement, but also for increasing the translational value of developmental neuroscience in this area (van Duijvenvoorde et al., 2016). Adopting this approach necessitates that we endeavor to make more precise predictions and use methods and samples that enable strong inferences about within-person change (Pfeifer and Allen, 2016). The present study attempts to rigorously test the adolescent social reorientation model in the context of evaluating oneself and others by implementing a greater level of developmental specificity and assessing change within individuals using a longitudinal design, as elaborated below.

### 1.1 Explicitly testing the adolescent social reorientation model

Perhaps because it is a broad-sweeping, heuristic model of social development, the social reorientation model has been widely applied to interpret neural and behavioral results that suggest adolescent-emergent or adolescent-specific patterns, across a variety of content domains and brain regions. A common approach is to contrast a peer versus non-peer context in a given brain region while participants, for example view faces (Saxbe et al., 2015), make economic (Braams et al., 2014, Braams and Crone, 2017, Smith et al., 2015, Smith et al., 2018) or risky driving decisions (Chein et al., 2011, van Hoorn et al., 2018), exert cognitive control (Breiner et al., 2018, Smith et al., 2018), evaluate themselves (Jankowski et al., 2014), or simply respond naturally (Somerville et al., 2013), and examine the developmental trajectory—often in cross-sectional samples. Then, peaks in early and/or middle adolescence, or differences between contexts in adolescent only samples, are described as being consistent with adolescent social reorientation and increased sensitivity to peers. This approach has provided substantial evidence verifying the plausibility of the model, but tells us relatively little about *how* social reorientation works. What specifically about peers is driving the effects across so many domains? Is it due to increased salience of information related to peer acceptance and integration as the model proposes? Are these changes unique to specific brain regions? When specifically are changes occurring? Do these changes differ as a function of domain? Careful consideration of the psychological processes, brain regions, developmental windows, and the reference conditions used to support inferences, will help support mechanistic refinement and development of neurocognitive and computational models of social reorientation (van den Bos and Eppinger, 2016).

More broadly, because adolescence is an extended stage of development lasting well over a decade (Dahl et al., 2018) during which myriad biological and social changes occur, it is necessary to define our hypotheses precisely so as to avoid over-generalizing our conclusions about both timing and processes (see Pfeifer and Allen, 2016). In order to characterize the degree and manner of social reorientation during early to middle adolescence, we need to precisely target both i) specific social changes that are highly developmentally salient at this time and ii) the appropriate hypothesis-driven neural processes/networks, as well as iii) use statistical modeling techniques that increase sensitivity and enable comparative anatomical hypothesis testing. By increasing precision in these areas, we can derive more specific hypotheses that represent stronger tests of the theoretical model (Pfeifer and Allen, 2016, van den Bos and Eppinger, 2016). We first briefly summarize evidence of two phenomena—social status and self-perception—that are considered highly salient and dynamic from early through middle adolescence. We then revisit the social information processing network proposed by Nelson and colleagues (2005, 2016) with an eye towards updating it to incorporate current understandings of neural systems for social processes, and discuss a novel modeling approach that increases sensitivity to detect developmental changes in these systems.

### 1.2 Salient social changes in adolescence

In some sense, a social reorientation towards peer relationships is readily observable in accounts of how adolescents spend their time: an increasing amount is spent with peers, beginning with school entry but accelerating across adolescence, particularly in mixed or opposite sex interactions (Lam et al., 2014, Larson and Richards, 1991). But what is particularly salient about these peer interactions during adolescence? There is a significant body of evidence suggesting an increased sensitivity to social status—which is an indicator of peer acceptance and integration—during early to middle adolescence (Crone and Dahl, 2012, LaFontana and Cillessen, 2010; see Dahl et al., 2018 for a review). For example, adolescents will prioritize social status feedback, even giving up potential monetary rewards in exchange (Cardoos et al., 2017). Some researchers have suggested that puberty-related changes in hormones such as testosterone, which is associated strongly with social status and dominance (Eisenegger et al., 2011), may make social status more salient during adolescence than at any other point in the lifespan (Dahl et al., 2018, Gardner and Steinberg, 2005). The increased use of relational aggression in adolescence also reveals the power of social status during this developmental stage, and some research suggests adolescents prioritize popularity over both prosocial behavior and academic achievements during early adolescence (de Bruyn and Cillessen, 2006, LaFontana and Cillessen, 2010). However, it is unclear whether these changes are driven by increases in the salience of social status, as suggested by the social reorientation model.

In addition, not all “reorientations” in adolescence are other-oriented; other prominent social cognitive changes during this period are self-focused (Crone and Fuligni, 2020). Multiple facets of self-perception change across adolescence, including self-esteem, self-complexity, self-concept clarity, self-consciousness, self-disclosure, and identity (Becht et al., 2016, Pfeifer and Allen, 2021, Pfeifer and Berkman, 2018, Somerville et al., 2013, Van der Cruijsen et al., 2019a, Van der Cruijsen et al., 2019b, Vijayakumar and Pfeifer, 2020)—all of which increase during adolescence except self-esteem, which is more nuanced (Baldwin and Hoffmann, 2002, Birkeland et al., 2012, Orth et al., 2018). A developmental approach posits that self-esteem represents a hierarchically organized summation of personal attributes and value across core domains such as academics, peer or family relationships, behavioral conduct, and physical appearance (Eccles et al., 1989, Harter, 2012). These specific self-concepts become increasingly complex and differentiated with age (Marsh, 1990a, Marsh, 1990b), due to acquisition of more social roles and relationships as well as greater specificity in academic subjects and other extracurricular activities. Consistent with the aforementioned findings of prioritizing social status over academic achievement, research often reveals “troughs” in academic self-concepts during early to middle adolescence (Cole et al., 2001, van der Cruijsen et al., 2018).

Of critical importance to this study, social status and self-perception intersect in the form of social self-concept, a domain-specific appreciation of one’s perceived social acceptance and competence (Berndt and Burgy, 1996). From childhood through early and middle adolescence, the social self-concept increasingly differentiates between family and peer contexts, including between same and opposite sex peers (Byrne and Shavelson, 1996). Most cross-sectional research suggests that social and academic self-concepts are unrelated, or only mildly positively associated; but longitudinal work suggests that the two become increasingly distinct over time, and that highly positive social self-concepts predict subsequent decreases in academic self-concepts from ages 10–14 (Preckel et al., 2013).

### 1.3 Beyond a neural social information processing network

The initial formulation of the social reorientation model identified three “nodes”—*detection*, *affective*, and *cognitive regulatory*—of a social information processing network implicated in the social changes during adolescence. At the time the model was proposed, these nodes represented basic phenomena for which something was known about the underlying neural processes such as biological motion, face perception, salience and threat detection, and cognitive regulation. In the intervening 15 years, one critical distinction researchers have made is between systems engaged in social cognition and those supporting cognitive regulation, rather than collapsing them into a single system. The former is frequently referred to as the “social brain” (Adolphs, 2009, Lieberman, 2007, Alcalá-López et al., 2018 for a recent process-oriented theory of the social brain) and includes multiple subregions of the medial prefrontal cortex (mPFC), medial posterior parietal cortex (mPPC), temporal-parietal junction (TPJ), and anterior temporal cortex (ATC). The social cognitive processes carried out by this network can be further specified as other-oriented (such as mentalizing about others’ dynamic beliefs in TPJ, and considering trait-like attributes of others in dorsal mPFC; Kliemann and Adolphs, 2018; Lieberman et al., 2019; Schurz et al., 2014) or self-focused (such as self-evaluation in ventral mPFC and perigenual ACC; Pfeifer and Berkman, 2018).

Developmental cognitive neuroscience research broadly confirms these functional mappings for the “social brain.” The same regions are implicated in the aforementioned suite of other-oriented and self-focused social cognitive processes—but the structure and function of these regions is known to change during adolescence (Barendse and Pfeifer, 2020, Blakemore, 2008, Fan et al., 2021, Kilford et al., 2016, McCormick et al., 2018, Mills et al., 2014, Pfeifer and Peake, 2012, Tamnes et al., 2017). One recent cross-sectional study looking at self-evaluation found that mPFC activation increased with age from 11 to 21 years, particularly for evaluations of physical appearance self-concept and less so for self-concepts in the academic and prosocial domains (van der Cruijsen et al., 2018). Focusing just on girls from ages 10–13, our own recent cross-sectional work observed no significant increases in social brain activity during evaluations of self-concepts in prosocial, antisocial, and social status domains (Barendse et al., 2020).

Because a system focused predominantly on social cognition was not discussed in the original (Nelson et al., 2005) or expanded (Nelson et al., 2016) social reorientation model, Nelson and colleagues did not make specific predictions about its relative engagement during adolescence. However, Nelson et al. (2016) emphasize that neural changes are highly dependent on the specific stimuli and task demands investigated. Therefore, in the context of evaluating oneself and others, we expect that activation of these other-oriented and self-focused cognition nodes should reflect the unique relevance of social status and self-perception during adolescence.

### 1.4 Increasing sensitivity to detect neurodevelopmental effects and test anatomical hypotheses

Although traditional brain mapping approaches have been used to reveal much of what we know about the neural underpinnings of self and social cognition, these approaches present particular challenges for developmental research generally, and for comparing developmental trajectories across a set of brain regions specifically. Task-based fMRI often suffers from low power (Turner et al., 2018) and this problem is magnified in developmental samples, which have greater heterogeneity in brain responses related to development, and are more likely to be contaminated with motion artifacts (Herting et al., 2018). In low-powered studies, when we adequately control for Type I errors using appropriate thresholding procedures, we cannot interpret the lack of activation clusters as evidence of no effect, because we are likely underpowered to detect one (Flournoy et al., 2020).

Furthermore, whole-brain analyses do not test interactions between brain regions (Jernigan et al., 2003), and thus cannot conclude that an effect is larger in one brain region than another, though researchers often make such interpretations. In other words, voxels that survive thresholding procedures are not necessarily significantly more active than voxels that do not. Whole-brain analyses are also often complemented with region of interest (ROI) analyses to test hypotheses about specific brain regions. Because a limited number of ROIs are typically used, it precludes detection of effects in other brain regions that were not selected as ROIs; because researchers rarely look at brain regions they do not expect to show effects, it constrains our ability to conclude that an observed effect in a given ROI is unique.

An alternative approach that addresses many of these limitations is to conduct hierarchical modeling with parcellated whole-brain data to directly test anatomical (i.e., spatial) hypotheses about a given set of brain regions compared to another. Parcellation retains the whole-brain nature of the data, but summarizes it into fewer data points by averaging across voxels within parcels. This results in a more tractable set of hundreds of parcels (rather than hundreds of thousands of voxels). From this data, researchers can identify a set of brain regions (e.g., social brain regions) of interest and directly test whether the observed relationships in this set differ from a control set (e.g., all other brain regions). This method also increases power to detect effects by pooling variance across parcels and individuals during hierarchical modeling. For these reasons, this approach is ideal for developmental neuroimaging studies testing spatial hypotheses and can enable stronger inferences than traditional brain-mapping methods.

### 1.5 Present study

In this study, we characterized developmental changes in the salience of social information that is expected to be highly relevant during early-to-mid adolescence. We aimed to 1) test a key prediction from the social reorientation model: that social information related to peer acceptance and integration, i.e., social status, becomes uniquely salient during adolescence, 2) examine the development of self-evaluation, and 3) test whether developmental effects are more prominent when status-related social information is self-relevant. We pursued these aims in the context of a 3-wave longitudinal functional magnetic resonance imaging (fMRI) study with adolescents ages 9–17 years. Although pubertal development is expected to drive shifts in the salience of social status (Dahl et al., 2018), the present longitudinal design sampled data only every 3 years on average. During a window of such duration, adolescents will normatively advance through 3 stages of puberty (Herman-Giddens, 2006), making our design suboptimal for examining social reorientation in direct association with puberty. However, we provide parallel models with puberty as the maturational index in Supplementary Material.

At each wave, participants completed a self/other evaluation task during which they made judgments about items related to social status or academic competence while being scanned. We focus on evaluation of oneself and others because figuring out who you are and how you fit in with your peers is a key developmental task during adolescence (Pfeifer and Peake, 2012). Since the self/other evaluation task could be considered a social judgment task that engages social cognition generally, this design allowed us to test whether developmental reorientation is unique to *status-related* social information by comparing it to another domain relevant during adolescence. We employed hierarchical growth curve modeling in order to test specific spatial hypotheses about brain regions associated with self and social cognition using parcellated whole-brain data with increased power. Using this approach, we examined the following research questions and hypotheses related to adolescent social reorientation:

1. **Does information about social status become more salient during adolescence?** According to the social reorientation model, social information related to peer acceptance and integration should become more salient during the transition from childhood into adolescence. Therefore, we expected to observe a linear and/or quadratic increase in blood-oxygenation-level-dependent (BOLD) signal for evaluation of social status relative to academic competence in brain regions associated with social cognition, as opposed to control regions. See Fig. 1 for hypothetical developmental trajectories that would be consistent with this hypothesis.

**Fig. 1:**
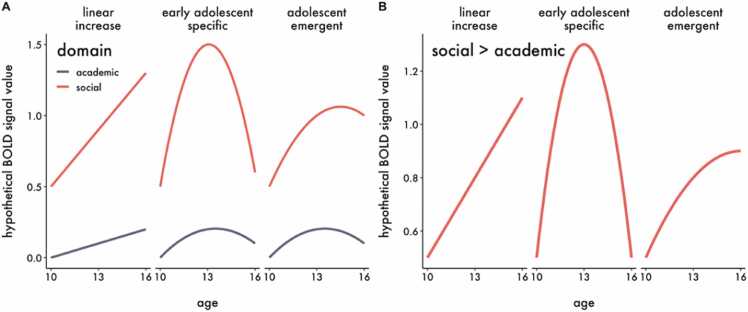
Hypothetical developmental trajectories for BOLD signal responses to A) status-related social and academic information, and B) status-related social > academic information, that would be consistent with Hypothesis 1 derived from the social reorientation model. These examples are illustrative, not exhaustive.
2. **Does information about the self become more salient during adolescence?** Given the dynamic changes in self-perception that occur in adolescence, we expected to observe a linear and/or quadratic increase in BOLD signal when evaluating information about the self relative to others in brain regions associated with self-referential processing compared to control regions.
3. **Are developmental changes in salience of social information moderated by evaluation target?** The social reorientation model does not consider whether social information is self-relevant. However, given the inherent salience of self-relevant information generally (Sui et al., 2015; reviewed in Humphreys and Sui, 2016) and changes in identity and self-concept development during adolescence specifically (Pfeifer and Berkman, 2018), we expected to observe enhancements of the developmental patterns (i.e., stronger linear or quadratic increases) A) described in Hypothesis 1 during self-evaluation, and B) described in Hypothesis 2 when evaluating information about social status.

## 2 Methods

### 2.1 Participants

**Fig. 2:**
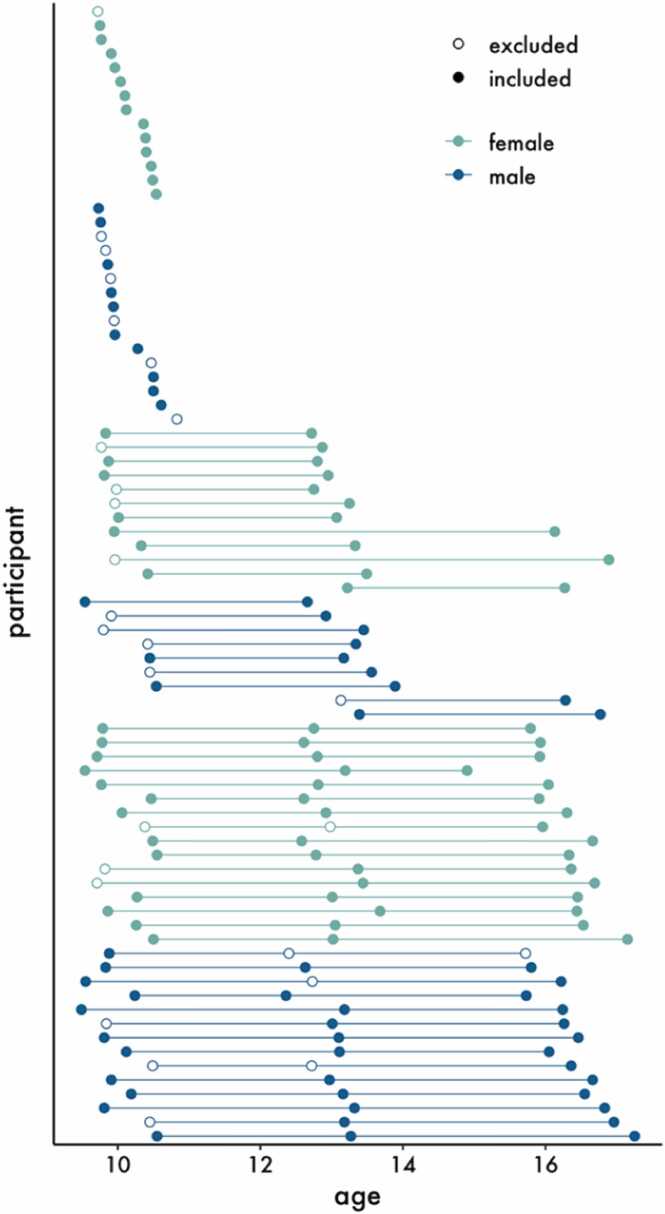
Age distribution of the sample across waves as a function of sex and whether or not they were included from the MRI analyses due to poor data quality or excessive motion. Thirty-five participants had one wave of data included in MRI analyses; 17 participants had two waves of data included; and 22 participants had 3 waves of data included.

Ninety participants (45 females) participated in a longitudinal project including up to three waves of data collection across six years. As described previously (Flournoy et al., 2016, Pfeifer et al., 2007, Pfeifer et al., 2011, Pfeifer et al., 2013, Vijayakumar et al., 2019), all participants had no history of psychiatric, neurological, or learning disorders at enrollment. Because this self/other evaluation task was a secondary part of a broader imaging protocol assessing language and emotion processing, not all participants who attended a session completed the self/other evaluation task in the MRI scanner. Of the 90 participants initially enrolled, 81 completed at least one wave of the self/other evaluation task; 78 completed the self/other evaluation task in the scanner at wave 1, 49 completed the task at wave 2, and 35 completed it at wave 3. Participants were excluded from neuroimaging analyses at each wave for excessive motion artifacts (*N*_*wave1*_ = 16, *N*_*wave2*_ = 1) and poor first-level data quality (*N*_*wave1*_ = 5, *N*_*wave2*_ = 4, *N*_*wave3*_ = 1), as described below. These exclusions yielded the following sample sizes for neural analyses at each wave: *N*_*wave1*_ = 57, *N*_*wave2*_ = 44, *N*_*wave3*_ = 34. Thirty-five participants had a single wave of data included in MRI analyses; 17 participants had two waves of data included; and 22 participants had 3 waves of data included (Table 1, Fig. 2). Demographic information is available in Supplementary Material (Table S1). This study was conducted at the University of California Los Angeles (UCLA) and approved by the UCLA Institutional Review Board. Adolescents and their parents provided written informed assent/consent and participants were compensated for their participation.

**Table 1:** Sample characteristics.

Wave	MRI inclusion status	*N*	*M*	*SD*	Female	Male
1	included	57	10.08	0.32	33	24
	excluded	21	10.07	0.33	8	13
2	included	44	13.06	0.33	25	19
	excluded	5	12.79	0.28	1	4
3	included	34	16.33	0.46	19	15
	excluded	1	15.72	–	–	1

### 2.2 Self/other evaluation task

As described previously (Pfeifer et al., 2007, Pfeifer et al., 2013), participants completed a self/other evaluation task in which they listened to short phrases in the Social (*n* = 20) or Academic domain (*n* = 20) and judged whether the phrases described themselves (“Self” condition) or a familiar fictional other, Harry Potter (“Other” condition). Phrases in the Social domain were related to social status, whereas phrases in the Academic domain were related to verbal academic competence. Within each domain, half of the phrases were positive (e.g., “I make friends easily”), and half were negative (e.g., “I make many spelling mistakes”), with Domain and Target orders counterbalanced and then randomly assigned. Participants made yes or no responses using a button box. In each of the four counterbalanced blocks (Self Social, Self Academic, Other Social, Other Academic), the phrases were presented auditorily, 3 s apart; each phrase lasted approximately 1 s. Blocks lasted 78 s and were separated by rest periods of 21 s. Behavioral results from this task and other self-reported measures of social and academic self-development are presented in Supplementary Material.

### 2.3 Neuroimaging data acquisition and preprocessing

Neuroimaging data were acquired on a Siemens Allegra 3 T scanner at the University of California, Los Angeles. For each participant, we acquired a high-resolution structural T2-weighted echo-planar imaging volume (spinecho, 36 axial slices, TR/TE = 5000/33 ms, matrix size = 128 x 128, FOV = 200 x 200 mm, 1.56 mm in-plane resolution, 36 slices, 3 mm thick) and a functional scan (gradient echo, TR/TE = 3000/25 ms, flip angle = 90°, matrix size = 64 x 64, FOV = 200 x 200 mm, 3.125 mm in-plane resolution, 36 slices, 3 mm thick). Stimuli were presented via high-resolution MRI-compatible goggles (Resonance Technology, Inc.).

DICOM images were converted to NIFTI format using MRIConvert (http://lcni.uoregon.edu/~jolinda/MRIConvert/) and skull-stripped using the Brain Extraction Tool from FMRIB's Software Library (FSL; http://www.fmrib.ox.ac.uk/fsl/). Skull-stripped images were then preprocessed using SPM12 (Wellcome Department of Cognitive Neurology; http://www.fil.ion.ucl.ac.uk/spm). Functional images were realigned to the participant mean image, and coregistered to the anatomical image. Coregistered images were then manually reoriented along the axis of the anterior and posterior commissure, spatially normalized to a T2-weighted Montreal Neurological Institute (MNI) standard, and resliced to 3 mm^3^. Resliced functional images were then smoothed using a 6 mm^3^ full-width at half maximum (FWHM) Gaussian smoothing kernel.

### 2.4 Univariate analyses

First-level statistical analyses were conducted using SPM12. Block-design condition effects were estimated using a fixed-effects general linear model and convolving the canonical hemodynamic response function with condition blocks. Separate regressors were entered for conditions of interest (Self Social, Self Academic, Other Social, Other Academic) and each condition block lasted 78 s. We also included five motion regressors of no interest. Realignment parameters were transformed into Euclidean distance and we included regressors for translation and rotation separately, as well as the displacement derivative of each.

Another “trash” regressor marked images with motion artifacts (e.g., striping) identified via automated motion assessment (https://github.com/dsnlab/social_reorientation/tree/main/mri/auto-motion) and visual inspection. Twenty-seven participant sessions were excluded from group-level analyses because they had more than 20% of volumes that included motion artifacts (*N* = 17) or poor data quality (e.g., visible striping, no motor or auditory activation) identified during first-level model quality control conducted by visual inspection of the contrast for all conditions > baseline (*N* = 10). Low frequency drift was removed using global scaling (consistent with Pfeifer et al., 2007, Pfeifer et al., 2013). Each condition and wave were estimated as separate contrasts versus baseline and used as inputs in second-level group analyses.

Second-level analyses were conducted using AFNI 3dLME (Chen et al., 2013), which utilizes voxel-level linear mixed effects modeling, in order to include all available timepoints for each participant. We regressed BOLD signal on the following fixed effects: evaluation Target (Self or Other), information Domain (Social or Academic), the linear effect of Age (centered at 13 years in the age model), the quadratic effect of Age (i.e., Age^2^), the interactions between Target and Domain, Target, Domain, and Age, and Target, Domain, and Age^2^. Participant intercepts and linear slopes of Age were treated as random effects. Contrast maps were generated for the following effects of interest:

Social > Academic

Social > Academic ✕ Age

Social > Academic ✕ Age^2^

Self > Other

Self > Other ✕ Age

Self > Other ✕ Age^2^

Target ✕ Domain

Target ✕ Domain ✕ Age

Target ✕ Domain ✕ Age^2^

To correct for multiple comparisons, cluster-extent thresholding was implemented using AFNI (Version 18.2.04; Cox, 1996). In accordance with recent guidelines (Cox et al., 2017), the spatial autocorrelation function was first estimated for each participant and wave separately using AFNI’s 3dFWHMx, and then averaged across subjects. To determine probability estimates of false-positive clusters given a random field of noise, Monte-Carlo simulations were conducted with AFNI’s 3dClustSim using the average autocorrelation function across subjects (ACF_age_ = 0.61, 4.64, 11.30). For both models, a voxel-wise threshold of *p* < .001 and cluster extent of *k* > 30 was estimated (voxel dimensions = 3 x 3 x 3 mm) to achieve a whole-brain cluster-wise familywise error rate of α = 0.05.

### 2.6 Parcellation analyses

#### 2.6.1 Parcel definition and parameter extraction

**Fig. 3:**
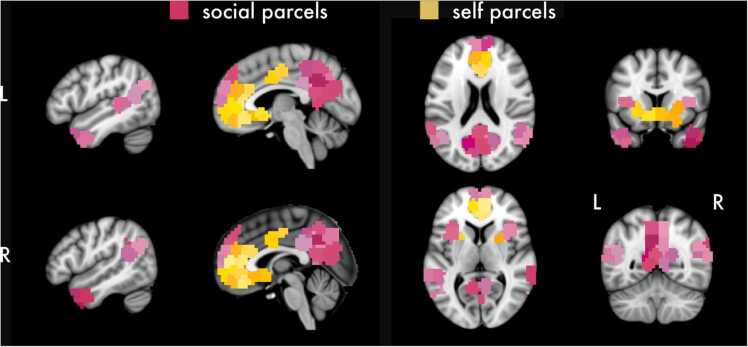
Whole-brain parcellation. Parcels from the Craddock 400 atlas were labeled as either related to self-evaluation, social processing, or control regions. For clarity, only social and self parcels are visualized. All other parcels were labeled as control regions and are available on Neurovault (https://neurovault.org/collections/SSEIPSAJ). Differences in color are used to distinguish individual parcels.

**Fig. 4:**
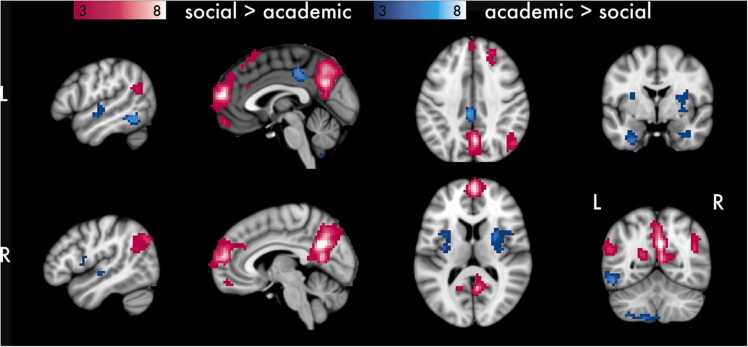
Univariate main effects of Domain collapsed across age. Results are thresholded at *p* < .001 and *k* = 30. Cluster extent (*k*) is measured in 3 x 3 x 3 mm voxels.

**Fig. 5:**
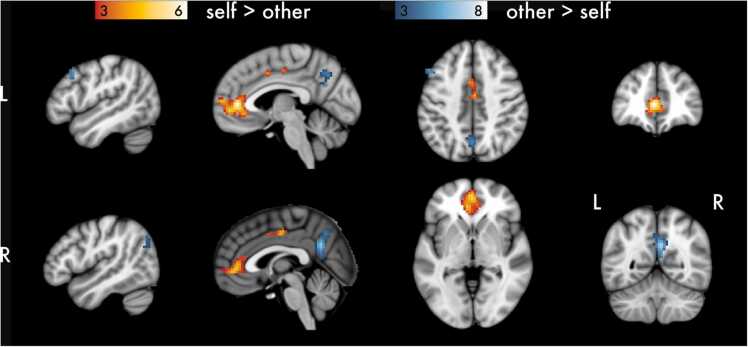
Univariate main effects of Target collapsed across age. Results are thresholded at *p* < .001 and *k* = 30. Cluster extent (*k*) is measured in 3 x 3 x 3 mm voxels.

We divided the brain into 352 parcels using the Craddock 400 parcellation atlas (Craddock et al., 2012). We selected this atlas because the size of the parcels were similar sized to activation clusters typically observed in task-based fMRI. Each parcel was categorized by authors Cosme, Pfeifer, and Livingston as being either related to self-evaluation (“self parcels,” *N* = 19) or social processing (“social parcels,” *N* = 44), or unrelated (“control parcels,” *N* = 289). Categorization of self and social parcels was based on the univariate main effects contrasts (i.e., collapsing across age), the association test meta-analytic maps (terms: “self referential” and “social”) from NeuroSynth (retrieved 2017; Yarkoni et al., 2011), and qualitative integration of the research literature (see also Denny et al., 2012; Lieberman et al., 2019; Pfeifer and Peake, 2012). Since we were interested in developmental effects in brain regions sensitive to self and social information in this sample, the univariate main effects contrasts strongly informed categorization. Consequently, some brain regions that are associated with both self-evaluation and social cognition (e.g., posterior cingulate cortex, precuneus) were categorized as social parcels because they were more responsive to other-oriented cognition in this sample (Fig. 4, Fig. 5). Given the frequent overlap between brain regions involved in self and social processing (Crone and Fuligni, 2020), we also created an interactive tool that can be used to explore how the developmental trajectories reported in this manuscript would change if a given parcel was recategorized (https://dcosme.shinyapps.io/growth_curves/). We included parcels unrelated to self and social processing in order to provide a control comparison when modeling. Control parcels were defined as all other parcels not classified as being related to self or social processing. We selected this approach, for several reasons. First, there are multiple brain networks that could serve as controls (e.g., visual, sensorimotor, or frontoparietal control networks) and no single brain network was clearly more appropriate (though for sensitivity analyses using sensorimotor or frontoparietal regions as controls showing the same pattern of results, see https://dsnlab.github.io/social_reorientation/analysis/sensitivity_analysis_controls). Second, selecting a single brain network would reduce the amount of data available for partial pooling during model estimation. Third, a single network may strongly influence the results if parcels within the network are sensitive to the task conditions making inferences difficult to draw. Finally, although it is possible to define the control parcels using different brain networks, estimate the model for each, and compare the results using e.g., specification curve analysis (Flournoy et al., 2020), this would be computationally intensive and would reduce power because less data is included. Self and social parcels are visualized in Fig. 3. All parcels maps, including the control regions, are available on NeuroVault (https://neurovault.org/collections/SSEIPSAJ).

For each first-level simple contrast (condition versus baseline), participant, and wave, we extracted the mean parameter estimate of BOLD signal within each parcel using the 3dmaskave function in AFNI 18.2.04 (Cox, 1996). Parameter estimates were standardized within parcel by dividing by the standard deviation across participants to account for differences in variability between parcels. Parcel outliers that were > 3 *SD* from the grand mean (0.79% of parcellation observations, *N* = 1503) were excluded.

#### 2.6.2 Multilevel model specification and comparison

We specified two cross-classified multilevel polynomial growth models (see equations) to test hypotheses from the social reorientation model. In each model, BOLD signal in each parcel was the criterion variable. In age models, age was centered at 13 years. Observations were nested within participants and parcel, and effects were allowed to vary across participant, parcel, or both. Random effects were selected so that the models would be as maximally unconstrained as possible in order to improve generalizability while still enabling model convergence (Barr et al., 2013). To facilitate interpretation of the models, factor levels were dummy coded as − 0.5 (Academic and Other) and 0.5 (Social and Self) so that the intercept represents the average across factor levels. Models were estimated in R 3.6.3. (R Core Team, 2018; https://www.r-project.org/) using the lmer function from the lme4 package (Version 1.1–25; Bates et al., 2015).

In the first model (“Model 1 – Domain”), we assessed whether the salience of social stimuli increases (either linearly or quadratically) across adolescence relative to academic stimuli, and whether developmental trajectories are unique to brain regions associated with social processing (i.e., whether they differed from trajectories in self or control parcels). We regressed BOLD signal from the simple contrasts on the following fixed variables: Domain (Social or Academic), Parcel Label (Self, Social, or Control), Age, Age^2^, and all nested interactions between 1) Domain, Parcel Label, and Age; and 2) Domain, Parcel Label, and Age^2^. This allowed us to estimate a fixed effect age trajectory for each cell of the design (Domain x Parcel Label). The Intercept, and the effects of Domain, Age, and their interaction were allowed to vary randomly across participants. The intercept, Domain, Age, and Age^2^ were modeled as random effects across parcels. We removed interactions between these variables as random effects so that the models would converge.

In the second model (“Model 2 – Domain x Target”), we tested whether these three-way interactions were moderated by Target (Self or Other). We included all nested interactions between the fixed effects of 1) Domain, Target, Parcel Label, and Age; and 2) Domain, Target, Parcel Label, and Age^2^. The main effect of Target was included as a random effect across participants and parcels, and the interactions between it and the other random effects specified in Model 1 – Domain were also treated as random across participants. Models were compared using the Akaike Information Criterion (AIC). A decrease in AIC of at least 2 points was considered to be a better fitting model. For equivalently fitting models, the more parsimonious model was selected for interpretation.

##### First level equations

Model 1 – Domain:Y_ijk_ = β_0jk_ + β_1jk_Age_ij_ + β_2jk_Age^2^_ij_ +

β_3jk_Domain_i_ +

Age_ij_ (β_4jk_Domain_ij_) +

Age^2^_ij_ (β_5jk_Domain_ij_) + ε_ijk_

Model 2 – Domain x Target:Y_ijk_ = β_0jk_ + β_1jk_Age_ij_ + β_2jk_Age^2^_ij_ +

β_3jk_Domain_ij_ + β_6jk_Target_ij_ +

β_7jk_Target_ij_Domain_ij_ +

Age_ij_ (β_4jk_Domain_ij_ + β_8jk_Target_ij_) +

Age^2^_ij_ (β_5jk_Domain_ij_ + β_9jk_Target_ij_) +

Age_ij_ (β_10jk_Domain_ij_Target_ij_) +

Age^2^_ij_ (β_11jk_Domain_ij_Target_ij_) + ε_ijk_

##### Second level equations

In Models 1 and 2:

β_0jk_ = γ_000_ + γ_001_Parcel Label_k_ + µ_00j_ + µ_00k_

β_1jk_ = γ_100_ + γ_101_Parcel Label_k_ + µ_00j_ + µ_10k_

β_2jk_ = γ_200_ + γ_201_Parcel Label_k_ + µ_20k_

β_3jk_ = γ_300_ + γ_301_Parcel Label_k_ + µ_30j_ + µ_30k_

β_4jk_ = γ_400_ + γ_401_Parcel Label_k_ + µ_40j_ + µ_40k_

β_5jk_ = γ_500_ + γ_501_Parcel Label_k_ + µ_50k_

In Model 2:

β_6jk_ = γ_600_ + γ_601_Parcel Label_k_ + µ_60j_ + µ_60k_

β_7jk_ = γ_700_ + γ_701_Parcel Label_k_ + µ_70j_ + µ_70k_

β_8jk_ = γ_800_ + γ_801_Parcel Label_k_ + µ_80j_ + µ_80k_

β_9jk_ = γ_900_ + γ_901_Parcel Label_k_ + µ_90k_

β_10jk_ = γ_1000_ + γ_1001_Parcel Label_k_ + µ_100j_ + µ_100k_

β_11jk_ = γ_1100_ + γ_1101_Parcel Label_k_ + µ_110k_

for *i* observations, *j* participants, and *k* parcels.

## 3 Results

### 3.1 Univariate analyses

To investigate brain regions that were associated with processing of social and academic information, we contrasted BOLD signal between the Social and Academic conditions collapsed across Age and Target (Fig. 4). Social information was associated with relatively stronger clusters of activation in anterior medial orbitofrontal cortex, posterior cingulate cortex and precuneus, and right superior frontal gyrus, as well as in regions associated with social processing, including bilateral anterior dmPFC and temporal parietal junction. Academic > Social phrases were associated with clusters of activation in a more rostral aspect of posterior cingulate cortex, bilateral claustrum, and right parahippocampus.

Collapsing across Domain and Age, Self evaluation was associated with relatively greater activation than Other evaluation in cortical midline structures implicated in self-focused cognition (Fig. 5). We observed a large cluster in mPFC, peaking in pgACC, and a smaller cluster in mid-cingulate cortex. Other evaluation was associated with relatively greater BOLD signal than Self evaluation in precuneus, left middle frontal gyrus, and right middle temporal gyrus. See Table 2 for all clusters of activation and relevant statistics.

**Table 2:** Regions, MNI coordinates, cluster extent, and peak Z values for contrasts of interest.

Contrast	Region	MNI Coordinates (x, y, z)			Extent (*k*)	Peak *Z*
*Social > Academic*	Precuneus	3	-61	29	652	9.13
	dmPFC	3	56	14	483	8.58
	R SFG	21	32	50	241	6.95
	R MTG	48	-70	35	171	5.65
	L STG	-54	-58	23	112	5.54
	mSFG	-3	26	59	74	4.64
	L PCC	-18	-58	17	46	5.49
	avmPFC	3	56	-16	40	5.02
	L MFG	-36	8	62	33	4.32
*Academic > Social*	R Claustrum	27	8	14	249	4.94
	L Claustrum	-33	-13	11	154	4.74
	L ITG	-54	-52	-13	113	6.56
	L Uncus	-36	-10	-31	98	5.62
	L Cerebellum VIIIb	-15	-58	-58	95	4.55
	pMCC	-3	-31	41	91	7.29
	R Parahippocampus	39	-40	-7	40	4.02
	R STG	48	-16	-4	40	4.17
	R Uncus	36	-7	-34	33	4.86
	L STG	-51	-10	-10	33	4.75
*Self > Other*	pgACC	-3	41	5	267	6.04
	MCC	3	-10	41	41	4.51
*Other > Self*	PCC	6	-55	26	147	5.43
	MTG	51	-70	26	38	4.25
	MFG	-51	20	44	38	4.38

*Note*. Cluster family-wise error correction for α = 0.05 and *p* < .001 is *k* = 30. Cluster extent (*k*) is measured in 3 x 3 x 3 mm voxels.

With respect to interactions among Domain, Target, and the linear and quadratic effects of Age, no clusters of activation survived thresholding in these contrasts. All unthresholded contrasts are available online (https://neurovault.org/collections/SSEIPSAJ).

### 3.2 Parcellation analyses

**Fig. 6:**
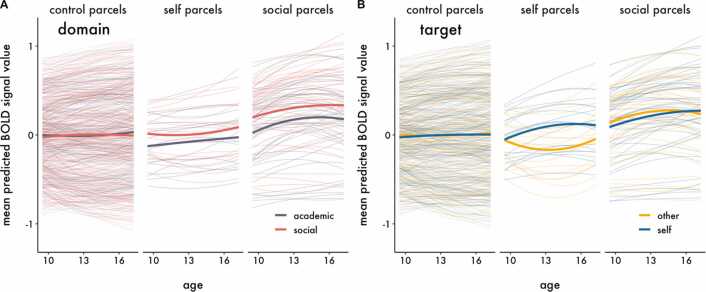
Predicted BOLD signal response from the best fitting model showing the developmental trajectories for the main effects of A) Domain and B) Target for each parcel label. Thin lines represent the predicted polynomial age effects for each parcel and condition; thick lines represent the mean developmental trajectory across parcels within each label and condition. Panel A shows that social parcels responded more strongly to social compared to academic information on average across adolescence. Panel B shows that self parcels responded more strongly to self compared to other evaluation on average across adolescence, though the magnitude of the difference varied by age.

**Fig. 7:**
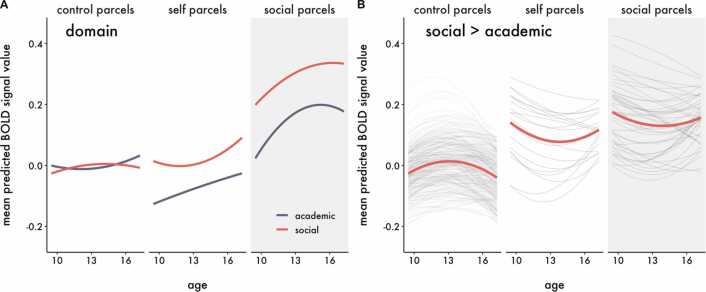
Predicted BOLD signal response from the best fitting model showing the developmental trajectories for A) Social and Academic information separately, and B) Social > Academic information for each parcel label collapsed across Target. Panel A visualizes the mean across parcels for each condition, magnified from Fig. 6A to better illustrate the developmental trajectories. Within social parcels, BOLD signal responses to Social and Academic information show largely the same developmental trajectories. Panel B shows that within social parcels, the difference in responses between Social and Academic follows a shallow U-shaped trajectory–decreasing in early adolescence, then increasing in mid-adolescence. Thin lines represent the predicted polynomial age effects for each parcel; thick lines represent the mean developmental trajectory across parcels within each label. These relationships in self parcels are provided for completeness. The primary parcel label of interest is highlighted in gray; the others are provided for completeness.

**Fig. 8:**
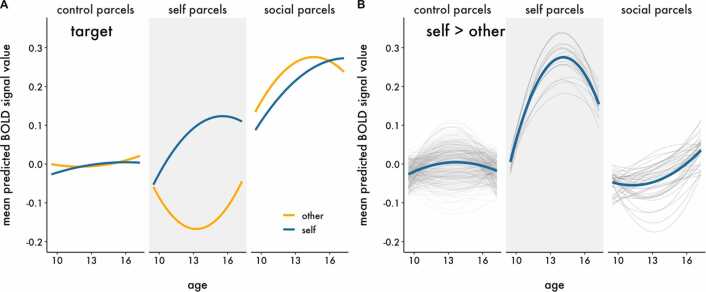
Predicted BOLD signal response from the best fitting model showing the developmental trajectories for A) Self and Other evaluation separately, and B) Self > Other evaluation for each parcel label collapsed across Domain. Panel A visualizes the mean across parcels for each condition, magnified from Fig. 6B to better illustrate the developmental trajectories. Within self parcels, BOLD signal responses to Self evaluation increase across adolescence, whereas they follow a U-shaped trajectory for Other evaluation. Panel B shows that within self parcels, the difference in responses between Self and Other follows an inverted U-shaped trajectory–increasing in early adolescence, then decreasing in mid-adolescence. Thin lines represent the predicted polynomial age effects for each parcel; thick lines represent the mean developmental trajectory across parcels within each label. The primary parcel label of interest is highlighted in gray; the others are provided for completeness.

**Fig. 9:**
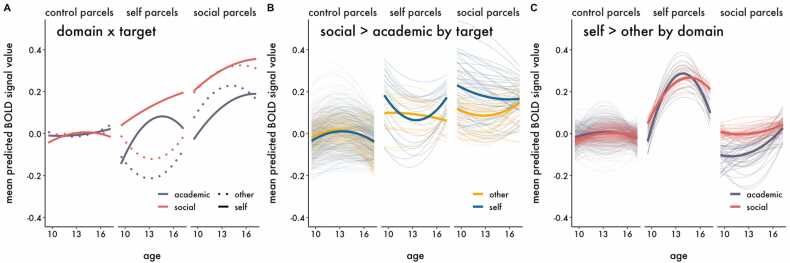
Predicted BOLD signal response from the best fitting model showing the developmental trajectories for the interaction between Domain and Target for each parcel category. Panel A visualizes the mean trajectory across parcels for each task condition separately, whereas Panel B shows the contrast of Social > Academic information as a function of Target and Panel C shows the contrast of Self > Other evaluation as a function of Domain, for each parcel. Thin lines represent the predicted polynomial age effects for each parcel; thick lines represent the mean developmental trajectory across parcels within each label. Panel A shows the positive linear slopes of BOLD signal responses to Social information during Self evaluation in both self and social parcels. Panel B shows that the difference in BOLD responses for Social > Academic information during Self evaluation decreases across adolescence in social parcels. Panel C shows that the difference in BOLD responses for Self > Other evaluation shows a weaker deceleration in mid-adolescence for Social information compared to Academic information.

Although no clusters of activation survived thresholding for the interactions in the univariate models, we cannot conclude that there are no true underlying interactions because we may be underpowered to detect them (Flournoy et al., 2020). In order to increase power and directly test our spatial hypotheses, we complemented the univariate analyses by using hierarchical growth curve modeling with parcellated whole-brain data. As described in the methods, we classified parcels as being related to either social processing (“social parcels”) or self-evaluation (“self parcels”), and assigned all remaining parcels as controls (“control parcels”). We regressed BOLD signal within parcels on predictors and compared two statistical models assessing the developmental trajectory of neural responses to evaluation of social and academic information within each class of parcels, collapsed across Target (Model 1 – Domain) and moderated by Target (Model 2 – Domain x Target), and found that Model 2 best fit the data (Table 3). The statistics interpreted below and presented in Table 5. While each hypothesis is tested by specific interactions highlighted in Table 5, lower order interactions are described as context to aid interpretation. The fitted parameter estimates in Fig. 6, Fig. 7, Fig. 8, Fig. 9 are from Model 2. Parallel figures with the raw data are provided in Supplementary Material. A sensitivity analysis estimating only the linear effect of age (and related interaction terms), is available online (https://dsnlab.github.io/social_reorientation/analysis/sensitivity_analysis_linear). The results from this analysis are consistent with those reported below, showing that the linear slope of age: (H1) did not differ for social versus academic items in social compared to control parcels, (H2) increased more strongly for self versus other evaluation in self compared to control parcels, (H3A) did not differ for self-evaluation of social versus academic items in social compared to control parcels, and (H3B) increased more strongly for self compared to other evaluation of social items in self compared to control parcels.

**Table 3:** Comparison of parcellation polynomial growth models.

Model	Model *df*	AIC	ΔAIC
1 – Domain	35	462224.6	
**2 – Domain x Target**	**151**	**461839.9**	**-384.7**

*Note*. The best fitting model is bolded. An AIC difference of at least 2 points was used as the criterion for a better fitting model.

**Table 4:** Results of the best fitting multilevel model with BOLD signal as the criterion.

Term	Fixed effects	*b* [95% CI]	*t*	*df*	*p*
γ_000_	Intercept (control label, age 13)	-0.004 [− 0.058, 0.050]	-0.14	380.66	.887
γ_100_	Age	0.003 [− 0.002, 0.009]	1.17	146.12	.244
γ_200_	Age^2^	0.000 [− 0.001, 0.001]	0.22	577.25	.828
γ_300_	Domain	0.013 [− 0.012, 0.039]	1.04	139.12	.302
γ_600_	Target	0.004 [− 0.015, 0.023]	0.40	188.97	.690
γ_001.self_	Label (self)	-0.033 [− 0.247, 0.180]	-0.31	352.12	.759
γ_001.soc_	**Label (social)**	**0.242 [0.096, 0.388]**	3.25	352.07	**.001**
γ_400_	Age x Domain	0.000 [− 0.007, 0.008]	0.13	58.01	.895
γ_800_	Age x Target	0.002 [− 0.002, 0.007]	1.00	75.18	.323
γ_101.self_	Age x Label (self)	0.007 [− 0.008, 0.022]	0.91	352.49	.361
γ_101.soc_	**Age x Label (social)**	**0.018 [0.008, 0.029]**	3.47	352.02	**.001**
γ_500_	**Age**^**2**^**x Domain**	**-0.003 [− 0.005, − 0.001]**	-3.00	1434.41	**.003**
γ_900_	Age^2^ x Target	-0.002 [− 0.004, 0.000]	-1.81	959.63	.070
γ_201.self_	Age^2^ x Label (self)	0.001 [− 0.003, 0.006]	0.58	366.80	.564
γ_201.soc_	**Age**^**2**^**x Label (social)**	**-0.004 [− 0.007, − 0.001]**	-2.82	364.92	**.005**
γ_301.self_	Domain x Label (self)	0.066 [− 0.006, 0.138]	1.80	447.87	.072
γ_301.soc_	**Domain x Label (social)**	**0.118 [0.069, 0.167]**	4.71	445.66	**< 0.001**
γ_700_	Target x Domain	-0.006 [− 0.043, 0.030]	-0.33	137.30	.746
γ_601.self_	**Target x Label (self)**	**0.255 [0.192, 0.318]**	7.92	475.08	**< 0.001**
γ_601.soc_	**Target x Label (social)**	**-0.051 [− 0.094, − 0.008]**	-2.32	472.03	**.021**
γ_401.self_	Age x Domain x Label (self)	-0.006 [− 0.020, 0.008]	-0.86	1437.56	.389
γ_401.soc_	[H1] Age x Domain x Label (social)	-0.005 [− 0.014, 0.005]	-0.96	1428.29	.340
γ_801.self_	**[H2] Age x Target x Label (self)**	**0.026 [0.013, 0.039]**	3.84	3904.83	**< 0.001**
γ_801.soc_	Age x Target x Label (social)	0.006 [− 0.003, 0.015]	1.37	3877.74	.172
γ_1000_	Age x Target x Domain	0.003 [− 0.008, 0.013]	0.53	54.84	.602
γ_501.self_	Age^2^ x Domain x Label (self)	0.006 [− 0.000, 0.013]	1.88	3673.42	.061
γ_501.soc_	**[H1] Age**^**2**^**x Domain x Label (social)**	**0.005 [0.001, 0.010]**	2.33	3641.72	**.020**
γ_901.self_	**[H2] Age**^**2**^**x Target x Label (self)**	**-0.011 [− 0.018, − 0.004]**	-3.01	1444.41	**.003**
γ_901.soc_	Age^2^ x Target x Label (social)	0.004 [− 0.000, 0.009]	1.76	1432.56	.078
γ_1100_	Age^2^ x Target x Domain	-0.000 [− 0.004, 0.004]	-0.07	1743.88	.946
γ_701.self_	Target x Domain x Label (self)	-0.022 [− 0.134, 0.090]	-0.39	3796.01	.700
γ_701.soc_	**Target x Domain x Label (social)**	**0.098 [0.021, 0.174]**	2.50	3765.90	**.013**
γ_1001.self_	[H3B] Age x Target x Domain x Label (self)	-0.006 [− 0.032, 0.021]	-0.42	17643.19	.677
γ_1001.soc_	[H3A] Age x Target x Domain x Label (social)	-0.014 [− 0.031, 0.004]	-1.50	17526.37	.134
γ_1101.self_	[H3B] Age^2^ x Target x Domain x Label (self)	0.009 [− 0.005, 0.022]	1.25	10786.63	.212
γ_1101.soc_	[H3A] Age^2^ x Target x Domain x Label (social)	-0.001 [− 0.010, 0.008]	-0.29	10695.06	.772

*Note.* Degrees of freedom (*df*) were calculated using the Satterthwaite approximation. Random effects are reported in Supplementary Material. P-values were not adjusted for multiple comparisons. Terms that include soc = social parcels, self = self parcels; all other terms are for control parcels. The primary tests for Hypotheses 1–3 are denoted in brackets (e.g., [H1]) before the term.

**Table 5:** Simple slope contrasts.

Contrast	Parcel	Slope	*b*	*df*	*t*	*p*
Self (Social > Academic)	Social	Age	-0.010	910.55	1.43	.153
Self (Social > Academic)	Social	Age^2^	0.002	7651.76	0.53	.598
**Social (Self > Other)**	**Self**	**Age**	**0.027**	**1664.99**	**2.77**	**.006**
Social (Self > Other)	Self	Age^2^	-0.008	4879.37	1.75	.081

Note. Degrees of freedom (df) were calculated using the Satterthwaite approximation.

As expected, when collapsing across Target and age, social parcels showed stronger BOLD signal than control parcels for Social relative to Academic information (γ_301.soc_ = 0.118, 95% CI [0.069, 0.167], *p* < .001). Collapsing across Domain and age, self parcels showed stronger BOLD signal than control parcels for Self compared to Other evaluation (γ_601.self_ = 0.255, 95% CI [0.192, 0.318], *p* < .001). Together, these results suggest that our parcellation classification scheme was appropriate. The developmental trajectories of these relationships are visualized in Fig. 6.

#### 3.2.1 Hypothesis 1: Developmental trajectories of salience for social versus academic information

All relationships reported in this section are averaged across Target. Collapsing across Academic and Social domains, we observed an adolescent emergent increase in BOLD signal in social relative to control parcels, characterized by a linear increase at age 13 (γ_101.soc_ = 0.018, 95% CI [0.008, 0.029], *p* = .001) and moderate deceleration (γ_201.soc_ = −0.004, 95% CI [−0.007, −0.001], *p* = .005). Based on the social reorientation model, we would expect this developmental trend to be stronger for social information; however, the developmental trajectories for Social and Academic information were largely equivalent. To draw conclusions about this hypothesis, we focus on the 3-way interactions between Domain, Label (social), and the linear and quadratic effects of Age. The linear effect of Age in social compared to control parcels did not differ between Social and Academic information at age 13 (Fig. 7A; γ_401.soc_= −0.005, 95% CI [−0.014, 0.005], *p* = .340). As indicated by the significant interaction between Age^2^ and Domain in social parcels, there was a weaker deceleration for Social compared to Academic information. However, this effect was relatively small, possibly indicating an earlier plateau for responsivity to Academic information but not a qualitatively different trajectory (Fig. 7B; γ_501.soc_= 0.005, 95% CI [0.001, 0.010], *p* = .020). If we correct these tests for two comparisons (*p*s = 0.680 and.040, respectively), the pattern of results remains the same.

#### 3.2.2 Hypothesis 2: Developmental trajectories of self versus other evaluation

All relationships reported in this section are averaged across Domain. Collapsing across Self and Other evaluation targets, we observed a weak, non-significant, linear increase in BOLD signal across adolescence in self relative to control parcels (γ_101.self_ = 0.007, 95% CI [−0.008, 0.022], *p* = .361). However, we hypothesized that this developmental trajectory would differ for Self relative to Other evaluation. To draw conclusions about this hypothesis, we focus on the 3-way interactions between Target, Label (self), and the linear and quadratic effects of Age. As expected, BOLD signal responses for Self evaluation increased across adolescence in self parcels (Fig. 8A), and the difference between Self and Other evaluation changed across adolescence. Specifically, the difference followed an inverted U-shaped developmental trajectory (Fig. 8B), as indicated by the positive interaction between Target and Age (γ_801.self_ = 0.026, 95% CI [0.013, 0.039], *p* < .001) and negative interaction between Target and Age^2^ in self compared to control parcels (γ_901.self_ = −0.011, 95% CI [−0.018, −0.004], *p* = .003). If we correct these tests for two comparisons (*p* < .002 and *p* = .006, respectively), the pattern of results remains the same.

#### 3.2.3 Hypothesis 3: Moderation by evaluation target

Collapsing across age, we observed an interaction between Target and Domain in social relative to control parcels. As expected, Self evaluation of Social information was associated with greater BOLD signal in social parcels (γ_701.soc_ = 0.098, 95% CI [0.021, 0.174], *p* = .013). However, we did not observe a statistically significant interaction between Target and Domain in self parcels when collapsing across age (γ_701.self_ = −0.022, 95% CI [−0.134, 0.090], *p* = .700).

With respect to developmental trajectories, responses to Social information about the Self generally showed a positive linear trajectory (with some deceleration) across adolescence in both self and social parcels (Fig. 9A). However, none of the 3-way interactions between Domain, Target, and Age or Age^2^ were statistically significant in either social or self parcels (Table 4, γ_1001_ and γ_1101_ parameters; Fig. 9B-C). In other words, we found no differences in the age trajectory that was a function of both Target and Domain, and this did not differ across parcel labels. However, our hypotheses were more specifically focused on the developmental trajectories for the differences between responsivity for A) Social and Academic information about the Self in social parcels, and B) Self and Other evaluation in the Social domain in self parcels. To examine these more targeted hypotheses, we estimated the simple slopes for these relationships (i.e., the instantaneous slopes at age 13). For Hypothesis 3A—Self evaluation in social parcels—the linear effect of Age was less positive for Social compared to Academic information, but this relationship was not statistically significant (*b* = −0.010, *p* = .153; Fig. 9A, social parcels, solid lines). Regarding Hypothesis 3B—responsivity to Social information in self parcels—the linear effect of Age was more positive for Self compared to Other evaluation (*b* = 0.027, *p* = .006; Fig. 9A, self parcels, pink lines) and the quadratic effect of Age was less positive for Self compared to Other evaluation, though not statistically significant (*b* = −0.008, *p* = .081; Fig. 9A, self parcels, pink lines). All statistics are reported in Table 5. Correcting the *p-*values for multiple comparisons across the four tests for 3A and the four tests for 3B does not change the pattern of results; the linear effect of Age pertaining to 3B remains the only significant coefficient (*p* = .024).

## 4 Discussion

The current study tested a key prediction from the social reorientation model by examining longitudinal changes in the neural responses to social information related to peer acceptance and integration relative to another salient domain, across adolescence. We examined this in the context of a self and other evaluation task and therefore focused on the “social brain,” which we expected to be uniquely sensitive to developmental changes in the salience of information about social status. Although no clusters of activation for contrasts testing the interaction between information domain and age survived thresholding in the univariate analyses, hierarchical growth curve modeling using parcellated whole brain data increased sensitivity to detect developmental effects. This model did not support a core prediction derived from the social reorientation model in this context: that *status-related* social information would become uniquely salient during adolescence. Although we observed an adolescent-emergent increase in neural responses to status-related social information in brain regions associated with other-oriented social cognition (compared to control brain regions), responses in these brain regions showed a similar trajectory for academic information, suggesting that these developmental changes were not *unique* to status-related social information. We also examined developmental changes in the salience of self-relevant information. We observed an inverted U-shaped developmental trajectory for self compared to other evaluation in brain regions associated with self-focused cognition, and this developmental trajectory was more pronounced for evaluation of status-related social information. Together, these results qualify existing models of adolescent social reorientation, and highlight the multifaceted changes in self and social development during adolescence.

### 4.1 Trajectories in other-oriented social brain regions

First, we found that neural responses in other-oriented social brain regions increased linearly for both social and academic information during early to mid-adolescence during self and other evaluation. Since academic judgments could be considered a type of social judgment because peers influence beliefs about academics (Rambaran et al., 2017), this finding could be construed as being consistent with the social reorientation model. However, a riskier prediction and stronger test of the model asserts that the developmental trend should be accentuated for social information related to peer acceptance and integration specifically. However, the rate of change was only slightly less negative during mid adolescence for social status items relative to academic items. Therefore, while these data are consistent with the notion that social information is highly salient across the lifespan and increases during adolescence during self and other evaluation, they are inconsistent with a strong, *unique* increase in salience for status-related social information during early to mid-adolescence—a prediction one could reasonably derive from the model (Nelson et al., 2016). This finding is also consistent with previous studies explicitly testing the adolescent social reorientation model (versus interpreting results in light of it) that did not observe adolescent peaks in the salience of peer social acceptance evaluations (Gunther Moor et al., 2010) or adolescent faces (Morningstar et al., 2019). If not false negatives, these results combined suggest that 1) the mechanism underlying adolescent social reorientation is not increased salience of information related to peer acceptance and integration, 2) increases in salience are more context-dependent than expected (and therefore may be observed in other domains, but not in the context of self and other evaluation), or 3) there are individual differences (e.g., related to one’s own social status) that moderate the degree of information salience and obscure a main effect. Testing these hypotheses will help refine the model and we hope that researchers will adopt the modeling strategy outlined here to increase power and enable anatomical hypothesis testing in pursuit of this goal.

### 4.2 Trajectories in self-focused brain regions

Second, we observed an inverted U-shaped developmental trajectory for self compared to other-evaluation that was unique to brain regions associated with self-focused cognition. This result is consistent with research showing substantial self-concept development during adolescence (Becht et al., 2016, Harter, 2012, Meeus et al., 1999) and the primacy of self-relevant information in cognitive processing, more broadly (Humphreys and Sui, 2016, Klein and Kihlstrom, 1986, Rogers et al., 1977, Symons and Johnson, 1997). These changes in the salience of the self are consistent with the idea that identity is an important source of value during adolescence that can be leveraged to promote healthy decision-making (Pfeifer and Berkman, 2018).

Finally, status-related social information about the self was especially salient and showed a positive linear developmental trajectory in brain regions associated with self-focused cognition. Although the social reorientation model has not explicitly considered the social target, these results indicate that adolescence is an important period for *social* self-development. This finding may reflect the impact that social information has in shaping the self (Koban et al., 2017, Korn et al., 2012) and the degree to which adolescents are particularly attuned to peer feedback (Somerville et al., 2013), especially in regard to peer acceptance and integration. However, because the developmental trajectories for self-evaluation of social and academic information only began to diverge in middle adolescence and did not statistically differ, these results also highlight the salience of academic self-concept, particularly during early adolescence. This finding is in line with research showing changes in academic self-concept during the transition from elementary to middle school (Preckel et al., 2013).

### 4.3 Limitations and future directions

These findings should be interpreted in light of several limitations. First, the longitudinal sampling procedure employed did not allow us to distinguish between adolescent-specific and adolescent-emergent developmental trajectories which would have required a wider age span. We focused on early and middle adolescence because this is when the social reorientation from peer play in childhood is expected to occur. However, further characterization of the observed effects within a longitudinal sample spanning childhood to adulthood would help to clarify the specific period in adolescence during which social information related to peer acceptance and integration is expected to peak, as well as transition into the next reorientation toward romantic relationships, which is expected to occur in late adolescence (Nelson et al., 2016, Pfeifer and Allen, 2021). Second, the sampling rate of the longitudinal design does not enable precise estimation of growth trajectories between waves and consequently may have introduced non-linearities not truly present in the data; therefore the fixed effects including quadratic effects of age should be interpreted cautiously. Third, we focused specifically on social status as the type of social information to test predictions from the social reorientation model, relative to a rigorous comparator—academic competence—that becomes increasingly relevant during adolescence, we did not compare different types of social information implicated in the model (Saxbe et al., 2015). Directly comparing the developmental trajectories for salience of various sources of social information that are expected to be more or less relevant during adolescence (e.g., related to mother, peer play, peer acceptance and integration, or romantic intimacy), would provide a strong test of the expanded enumeration of the social reorientation model that incorporates a lifespan perspective (Nelson et al., 2016, Van der Cruijsen et al., 2019a, Van der Cruijsen et al., 2019b). Fourth, As is the case with the majority of research on self/other evaluation that does not use close others, participants did not personally know the other person and were therefore making guesses about their abilities and preferences, informed by what they had read and seen about them. Future research should take this into consideration by, for example, assessing self-referential processing in other contexts that do not rely on explicit evaluations. Fifth, because the social reorientation model is agnostic to valence and because of the blocked task design, we collapsed across stimulus valence. Given the connections between self-concept, self-esteem, and mental health, the role of valence is an important avenue for future study (Barendse et al., 2020, Silk et al., 2017, van der Cruijsen et al., 2018). Sixth, we focused on chronological age as the developmental marker but future studies utilizing a more appropriate sampling window should investigate changes in self and social development related to puberty. To facilitate this endeavor, we have included additional models with puberty as the maturational index—which are largely consistent with the age models reported in the main manuscript—in Supplementary Material. Seventh, although BOLD signal intensity change is frequently interpreted as a measure of salience and depth of processing, it is not a direct measure of salience. There is research that suggests this interpretation is warranted in this context (Humphreys and Sui, 2016), but this assumption is not directly addressed in this study. Finally, although we created an interactive app (https://dcosme.shinyapps.io/growth_curves/) to allow readers to examine how the results would change if self and social parcels were assigned to different categories, we did not conduct sensitivity analyses investigating the robustness of the results using different parcellation atlases because of computational costs.

### 4.4 Contributions

Despite these limitations, this study has several notable strengths. First, it fills an important gap because studies explicitly testing the adolescent social reorientation model often employ cross-sectional (Gunther Moor et al., 2010, Morningstar et al., 2019) rather than longitudinal designs. We used rigorous comparators within the task and parcel categories, which enabled us to test the uniqueness of the effects of interest, strengthening the inferences that can be drawn from this study. We also applied growth curve modeling to parcellated whole-brain neuroimaging data (similar to the method proposed by Chen et al., 2019). These cross-classified hierarchical models allowed us to test specific spatial hypotheses by categorizing parcels within theoretically meaningful categories and test the uniqueness of the effects in these regions against a set of control regions. This approach also increased power (Gelman et al., 2012) to detect developmental effects beyond standard univariate methods by partially pooling information across parcels and participants and adjusting in a model-driven way for multiple comparisons. Given that many functional neuroimaging studies are underpowered to detect developmental effects using traditional univariate methods (Flournoy et al., 2020), we hope that others will adopt this framework to enable more specific and robust developmental hypothesis testing. We also encourage researchers to explore other methods that can increase sensitivity, such as by leveraging information represented in patterns of brain responses through multivoxel pattern analysis (Weaverdyck et al., 2020). For example, future research could assess how decoding accuracy or similarity of status-related social information and other information changes across development within social brain regions.

### 4.5 Conclusions

Together, these methodological strengths enabled us to rigorously test the adolescent social reorientation model in the context of self and other evaluation. We found that the salience of social information increased across adolescence, but that this developmental trajectory was not unique to status-related social information, as predicted by the social reorientation model. We also found that self-relevant information—especially in the social domain—becomes increasingly salient during adolescence, providing a novel means of confirmation that self-concept development is also an important feature of adolescence. These findings have implications for adolescent health and well-being, as sensitivity to personally and socially relevant information could serve both as a potential risk factor and as an opportunity for positive youth development. Translational neuroscience can leverage these changes in salience to design more effective interventions to improve adolescent health and well-being (Horn et al., 2020).

## Declaration of Competing Interest

The authors declare that they have no known competing financial interests or personal relationships that could have appeared to influence the work reported in this paper.

## Data Availability

The code and data to reproduce these analyses are available on GitHub (https://github.com/dsnlab/social_reorientation), and the parcellation maps, unthresholded statistical maps from the univariate fMRI analyses, and parameter estimates from the growth curve models are available on NeuroVault (https://neurovault.org/collections/SSEIPSAJ; Gorgolewski et al., 2015). In developing the analytic approach using parcellated whole-brain data, we deviated from our initial analysis plan in order to more effectively test our hypotheses. For transparency, we link to our original analysis plan (https://osf.io/7z4jd/).
