# Supplementary material for: Testing the adolescent social reorientation model during self and other evaluation using hierarchical growth curve modeling with parcellated fMRI data
Source: Dev Cogn Neurosci. 2022 Feb 23;54:101089. doi: 10.1016/j.dcn.2022.101089 (PMC8891708; doi:10.1016/j.dcn.2022.101089)
Supplement: Supplementary file 1 — Supplementary material [file mmc1.docx]

**Supplementary Material**

Testing the adolescent social reorientation model during self and other evaluation using hierarchical growth curve modeling with parcellated fMRI data

Danielle Cosme

John C. Flournoy

Jordan L. Livingston

Matthew D. Lieberman

Mirella Dapretto

Jennifer H. Pfeifer

**Demographic information**

Table S1 contains the racial and ethnic composition of the sample.

| Table S1.  *Sample Race and Ethnicity* | | |
| --- | --- | --- |
| Race | Not Hispanic or Latina/Latino | Hispanic or Latina/Latino |
| Black or African American | 3.3% | – |
| Multiracial | 11.1% | 5.6% |
| Native American | 4.4% | 1.1% |
| Pacific Islander | 4.4% | 1.1% |
| White | 43.3% | 25.6% |

**Figures visualizing the raw data**

While we visualized the predicted values from the best-fitting growth curve model in the main text, here we visualize the developmental trajectories using the raw (unfitted) data.

**
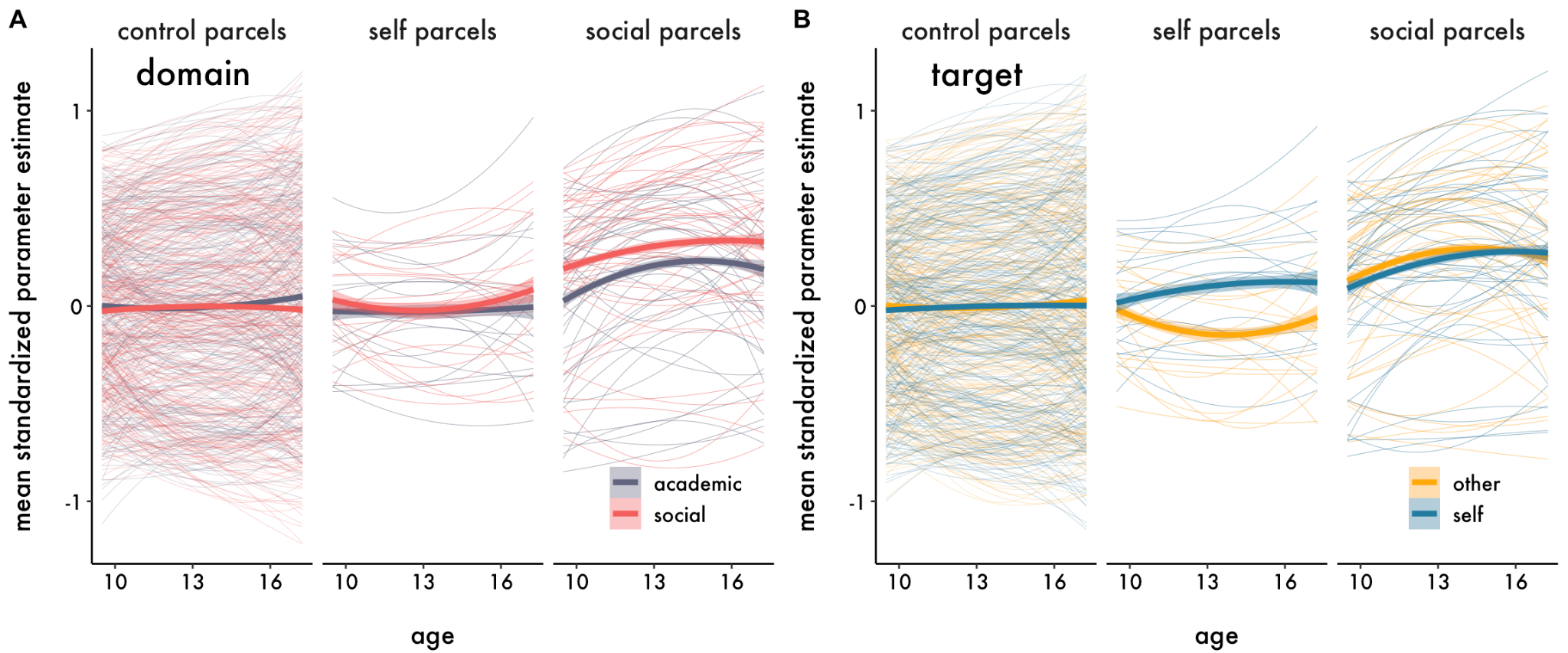
**

*Figure S1*. Raw BOLD signal response from the best fitting model showing the developmental trajectories for the main effects of A) Domain and B) Target for each parcel label. Thin lines represent the predicted polynomial age effects for each parcel and condition; thick lines represent the mean developmental trajectory across parcels within each label and condition. Growth curves were estimated using a quadratic function. Error bands are 95% confidence intervals


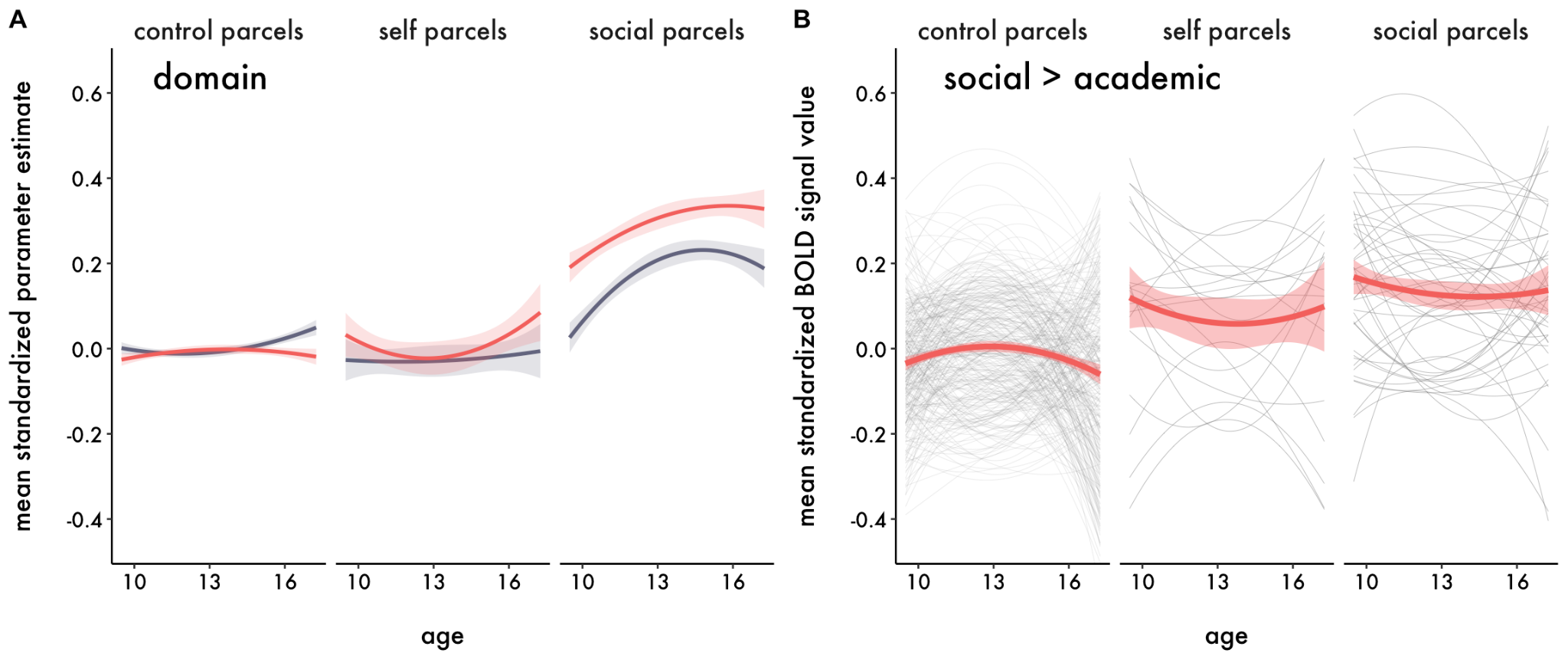


*Figure S2*. Raw BOLD signal response showing the developmental trajectories for A) Social and Academic information separately, and B) Social > Academic information for each parcel label collapsed across Target. Panel A visualizes the mean across parcels for each condition, magnified from Figure S1 to better illustrate the developmental trajectories. Thin lines represent the predicted polynomial age effects for each parcel; thick lines represent the mean developmental trajectory across parcels within each label. These relationships in self parcels are provided for completeness. Growth curves were estimated using a quadratic function. Error bands are 95% confidence intervals.


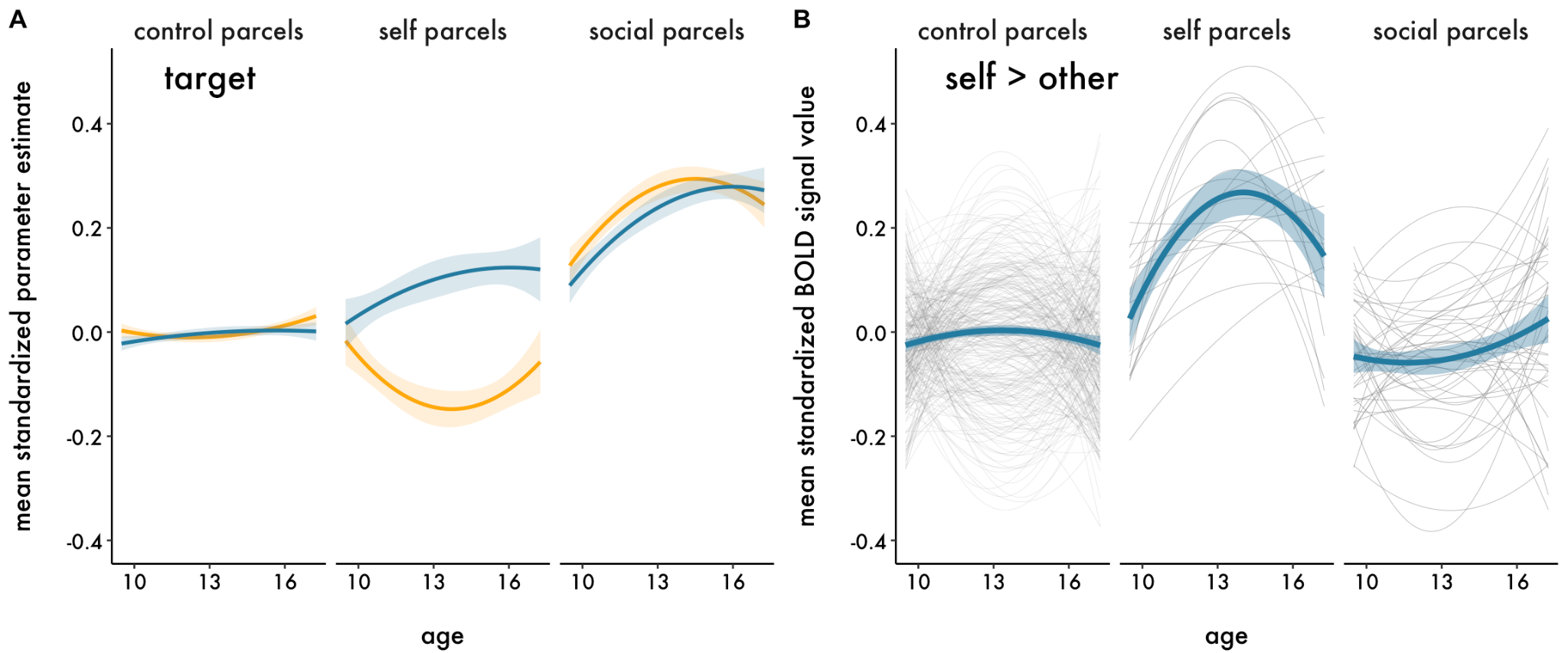


*Figure S3*. Raw BOLD signal response showing the developmental trajectories for A) Self and Other evaluation separately, and B) Self > Other evaluation for each parcel label collapsed across Domain. Panel A visualizes the mean across parcels for each condition, magnified from Figure S1 to better illustrate the developmental trajectories. Thin lines represent the predicted polynomial age effects for each parcel; thick lines represent the mean developmental trajectory across parcels within each label. These relationships in social parcels are provided for completeness. Growth curves were estimated using a quadratic function. Error bands are 95% confidence intervals.

*
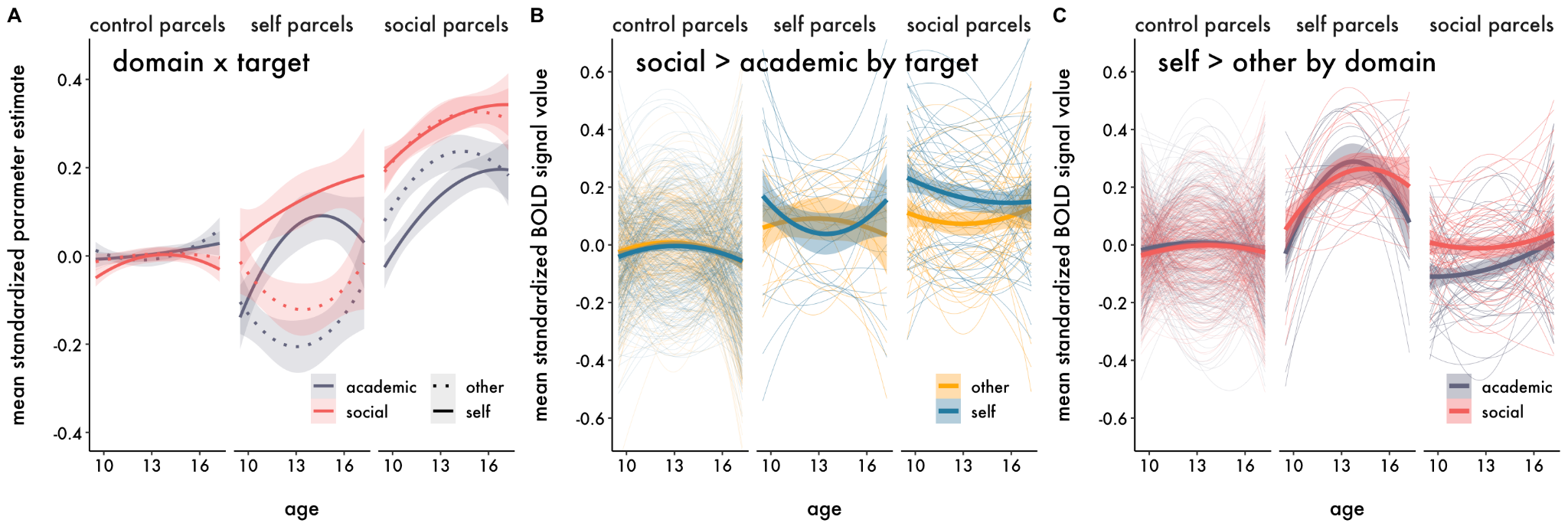
*

*Figure S4*. Raw BOLD signal response from the best fitting model showing the developmental trajectories for the interaction between Domain and Target for each parcel category. Panel A visualizes the mean trajectory across parcels for each task condition separately, whereas Panel B shows the contrast of Social > Academic information as a function of Target and Panel C shows the contrast of Self > Other evaluation as a function of Domain, for each parcel. Thin lines represent the predicted polynomial age effects for each parcel; thick lines represent the mean developmental trajectory across parcels within each label. Growth curves were estimated using a quadratic function. Error bands are 95% confidence intervals.

| Table S2  *Random effects from the Best Fitting Multilevel Model with BOLD Signal as the Criterion Reported in Table 5* | | | | | |
| --- | --- | --- | --- | --- | --- |
| Random effects | *SD* |  |  |  |  |
| Parcellation |  |  |  |  |  |
| Intercept | 0.456 |  |  |  |  |
| Age | 0.030 |  |  |  |  |
| Age^2^ | 0.006 |  |  |  |  |
| Target | 0.067 |  |  |  |  |
| Domain | 0.100 |  |  |  |  |
| Age x Target | 0.005 |  |  |  |  |
| Age^2^ x Target | 0.005 |  |  |  |  |
| Age x Domain | 0.010 |  |  |  |  |
| Age^2^ x Domain | 0.003 |  |  |  |  |
| Target x Domain | 0.049 |  |  |  |  |
| Age x Target x Domain | 0.005 |  |  |  |  |
| Age^2^ x Target x Domain | 0.003 |  |  |  |  |
| Participant |  |  |  |  |  |
| Intercept | 0.045 |  |  |  |  |
| Age | 0.014 |  |  |  |  |
| Target | 0.038 |  |  |  |  |
| Domain | 0.069 |  |  |  |  |
| Age x Target | 0.012 |  |  |  |  |
| Age x Domain | 0.023 |  |  |  |  |
| Target x Domain | 0.086 |  |  |  |  |
| Age x Target x Domain | 0.030 |  |  |  |  |
| Residual | 0.815 |  |  |  |  |
| *Note.* The correlations between the random effects can be accessed in the code repository for this project: <https://dsnlab.github.io/social_reorientation/analysis/main_analyses> | | | | | |

**Analyses with puberty as the maturational index**

Data collection in this longitudinal study occurred approximately every 3 years between the ages of 10-16 and is therefore not ideal for estimating developmental effects related to pubertal development, as noted in the main text. We include additional analyses using puberty as the maturational index here for completeness and to inform future studies testing puberty-related social reorientation in more appropriate samples.

**Pubertal development.** At each wave, pubertal development was assessed with the self-reported Pubertal Development Scale (PDS; Peterson et al., 1988). This scale consists of items that measure physical and body hair growth, skin changes, breast development and menarche in females, and voice changes and facial hair growth in males. Female participants responded “yes” or “no” to the menarche item; all other items were rated using a 4-point scale with the following anchors: “no physical changes” to “development seems complete.” This measure was scored using the Shirtcliff method (Shirtcliff et al., 2009) to approximate Tanner stage.

| Table S3  *Bivariate Correlations and 95% Confidence Intervals Among Age and Pubertal Stage*  *Bivariate Correlations and 95% Confidence Intervals Among Age and Pubertal Stage* | | | | | | |
| --- | --- | --- | --- | --- | --- | --- |
|  | Age 1 M | Age 2 M | Age 3 M | PDS 1 F | PDS 2 F | PDS 3 F |
| Age 1 F | – | – | – | .50*** | .20 | .01 |
| Age 2 F | – | – | – | -.12 | .40* | .30 |
| Age 3 F | – | – | – | -.11 | .02 | .24 |
| PDS 1 M | -.32* | -.37 | -.26 | – | .42* | -.08 |
| PDS 2 M | .22 | .41* | .50* | .16 | – | .25 |
| PDS 3 M | -.24 | .37 | .41 | .30 | .62** | – |
| *Note*. Correlations above the diagonal are for females; those below the diagonal are for males. The numbers (1-3) refer to the wave of data collection. M = male; F = female; PDS = pubertal stage scored using the Shirtcliff method. **p* < .05, ***p* < .01, ****p* < .001. | | | | | | |

**Multilevel modeling procedure.** We followed the same modeling procedure outlined in the main text, but with pubertal stage rather than age as the maturational index. Three participants were missing pubertal status at wave 1 and were therefore excluded from these analyses.


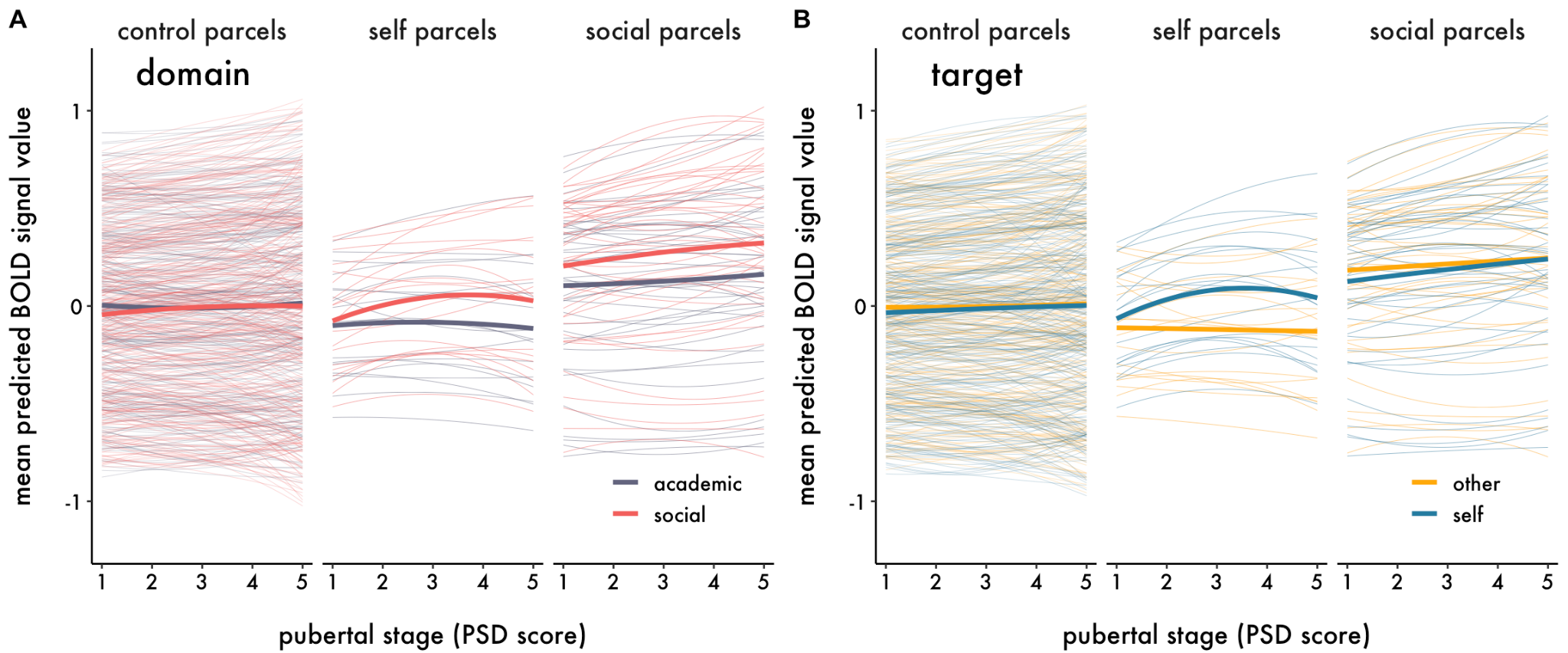


*Figure S5*. Predicted BOLD signal response from the best fitting puberty model showing the developmental trajectories for the main effects of A) Domain and B) Target for each parcel label. Thin lines represent the predicted polynomial puberty effects for each parcel and condition; thick lines represent the mean developmental trajectory across parcels within each label and condition. Panel A shows that social parcels responded more strongly to social compared to academic information on average across adolescence. Panel B shows that self parcels responded more strongly to self compared to other evaluation on average across adolescence, though the magnitude of the difference varied by puberty.

**Hypothesis 1: Developmental trajectories of social versus academic information.** All relationships reported in this section are averaged across Target. As expected, we observed stronger BOLD signal for Social relative to Academic information in social compared to control parcels when collapsed across puberty (Figure S5A; *b* = 0.144, 95% CI [0.099, 0.189], *p* < .001). Collapsed across Domain, we observed a linear increase in BOLD signal in social relative to control parcels across pubertal development, but this effect was not statistically significant (*b* = 0.015, 95% CI [-0.002, 0.032], *p* = .083). Based on the social reorientation model, we expected the developmental trend to be stronger for social information. However, the linear effect of Age in social compared to control parcels was not stronger for Social than Academic information (Figure S6B; *b* = 0.007, 95% CI [-0.013, 0.027], *p* = .498). Statistics and all model parameters can be found in Table S3.


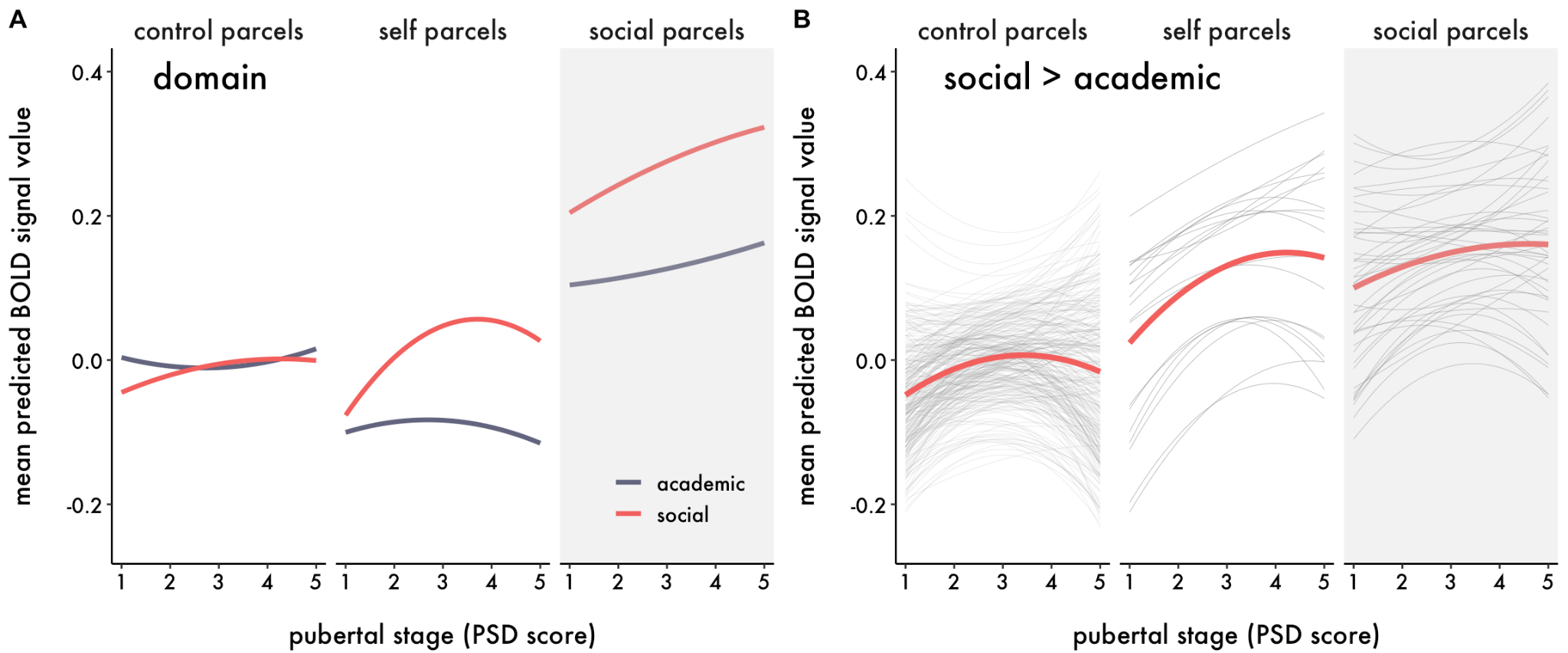


*Figure S6*. Predicted BOLD signal response from the best fitting puberty model showing the developmental trajectories for A) Social and Academic information separately, and B) Social > Academic information for each parcel label collapsed across Target. Panel A visualizes the mean across parcels for each condition, magnified from Figure S5 to better illustrate the developmental trajectories. Thin lines represent the predicted polynomial puberty effects for each parcel; thick lines represent the mean developmental trajectory across parcels within each label. These relationships in self parcels are provided for completeness.

**Hypothesis 2: Developmental trajectories of self relative to other evaluation.** All relationships reported in this section are averaged across Domain. As expected, self parcels had stronger BOLD signal during Self compared to Other evaluation, when collapsed across puberty (Figure 5B; *b* = 0.214, 95% CI [0.155, 0.273], *p* < .001). Across pubertal development, the difference between Self and Other showed an inverted U-shaped developmental trajectory (Figure S7B). Specifically, we observed a positive linear (*b* = 0.027, 95% CI [-0.002, 0.055], *p* = .070) and negative quadratic interaction (*b* = -0.022, 95% CI [-0.048, 0.005], *p* = .109) between Target and Puberty in self compared to control parcels. However, neither of these developmental trajectories were statistically significant in this model.


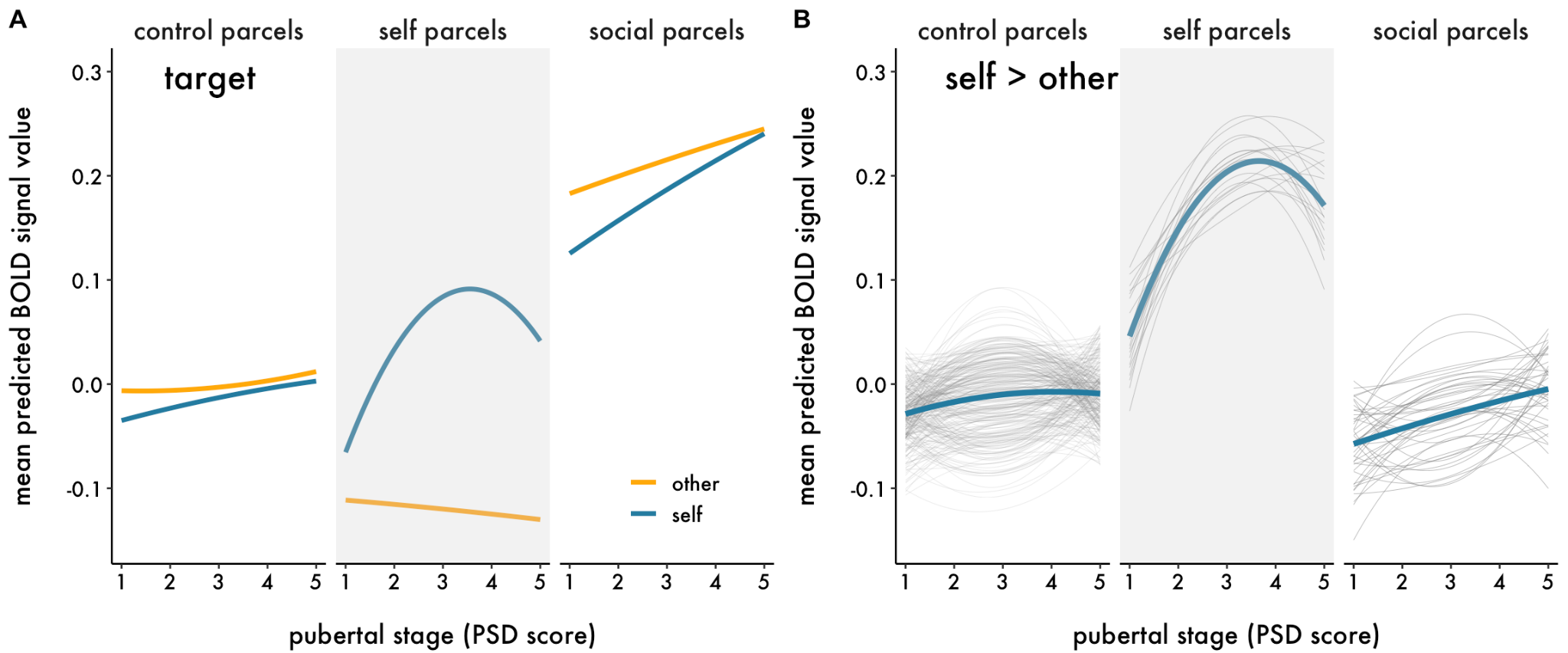


*Figure S7*. Predicted BOLD signal response from the best fitting puberty model showing the developmental trajectories for A) Self and Other evaluation separately, and B) Self > Other evaluation for each parcel label collapsed across Domain. Panel A visualizes the mean across parcels for each condition, magnified from Figure S5 to better illustrate the developmental trajectories. Thin lines represent the predicted polynomial puberty effects for each parcel; thick lines represent the mean developmental trajectory across parcels within each label. These relationships in social parcels are provided for completeness.

**Hypothesis 3: Moderation by evaluation target.** Social information about the self showed a positive linear trajectory across pubertal development in social parcels and a weakly inverted-U trajectory in self parcels (Figure S8A). We did not observe an interaction between Target and Domain in social relative to control parcels. The difference between evaluating Social and Academic information was associated with greater BOLD signal for Self compared to Other, but this effect was not statistically significant (*b* = 0.055, 95% CI [0.021, 0.131], *p* = .154). In self parcels, this interaction was in the opposite direction. Given the relative salience of academic information, the difference between evaluating Social and Academic information was associated with weaker BOLD signal for Self compared to Other (*b* = -0.129, 95% CI [-0.240, -0.017], *p* = .023).

With respect to differences in developmental trajectories in social parcels, the difference in BOLD signal between Social and Academic information actually decreased across pubertal developmental for Self relative to Other evaluation (Figure S8B; *b* = -0.025, 95% CI [-0.064, 0.014], *p* = .212). However, this interaction was not statistically significant. In self parcels, there was an interaction between Target, Domain, and the Puberty^2^. This developmental trajectory was largely driven by Self > Other evaluations for academic information which “peaked” in mid-puberty, whereas the difference between evaluation targets for social information showed a U-shaped trajectory in self parcels due to increased salience of information about others in the social domain (Figure S8C; *b* = 0.101, 95% CI [0.049, 0.153], *p* < .001).


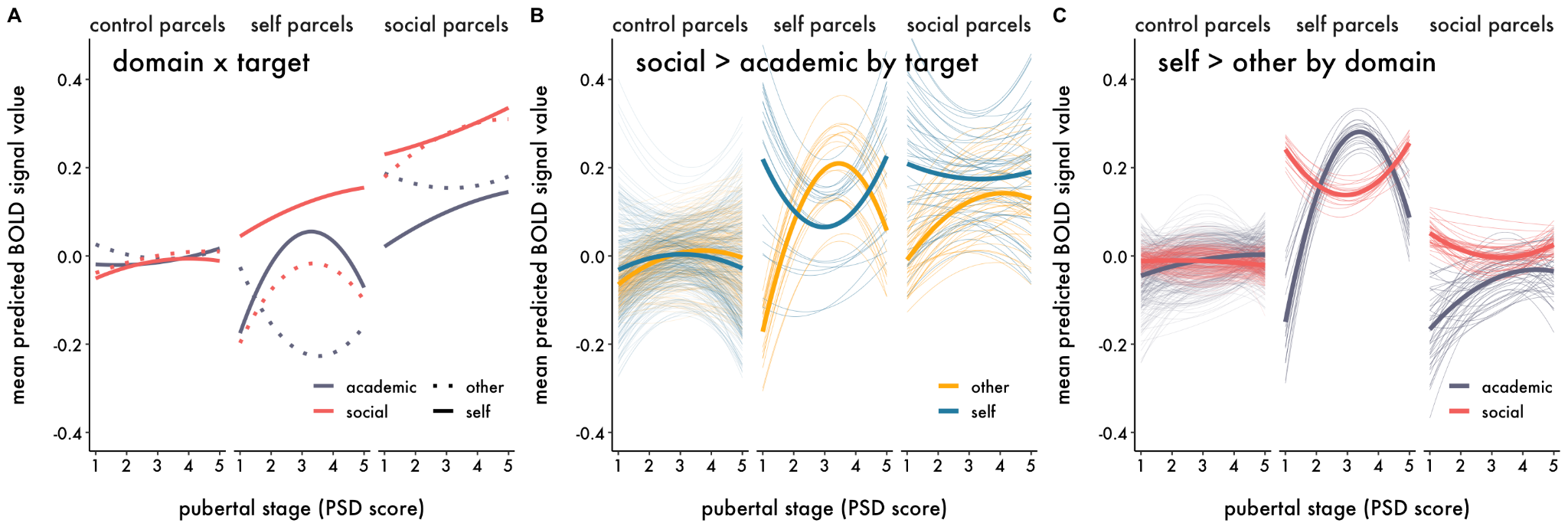


*Figure S8.* Predicted BOLD signal response from the best fitting puberty model showing the developmental trajectories for the interaction between Domain and Target for each parcel category. Panel A visualizes the mean trajectory across parcels for each task condition separately, whereas Panel B shows the contrast of Social > Academic information as a function of Target and Panel C shows the contrast of Self > Other evaluation as a function of Domain, for each parcel. Thin lines represent the predicted polynomial puberty effects for each parcel; thick lines represent the mean developmental trajectory across parcels within each label.

| Table S4  *Results of the Best Fitting Multilevel Model with BOLD Signal as the Criterion* | | | | |
| --- | --- | --- | --- | --- |
| Fixed effects | *b* [95% CI] | *t* | *df* | *p* |
| Intercept (control label, pubertal stage 3) | -0.008 [-0.060, 0.044] | 0.30 | 387.14 | .767 |
| Puberty | 0.007 [-0.003, 0.018] | 1.31 | 105.41 | .192 |
| Target | -0.010 [-0.028, 0.008] | 1.09 | 156.89 | .278 |
| Domain | 0.005 [-0.020, 0.030] | 0.39 | 120.91 | .695 |
| Label (self) | -0.010 [-0.215, 0.195] | 0.10 | 352.14 | .923 |
| **Label (social)** | **0.209 [0.069, 0.349]** | **2.93** | **352.05** | **.004** |
| Puberty^2^ | 0.000 [-0.005, 0.006] | 0.12 | 536.29 | .904 |
| Puberty x Target | 0.005 [-0.006, 0.016] | 0.89 | 48.63 | .376 |
| Puberty x Domain | 0.008 [-0.009, 0.025] | 0.91 | 60.67 | .366 |
| Target x Domain | -0.003 [-0.040, 0.034] | 0.15 | 143.73 | .880 |
| Puberty x Label (self) | 0.004 [-0.021, 0.029] | 0.31 | 354.58 | .754 |
| Puberty x Label (social) | 0.015 [-0.002, 0.032] | 1.74 | 354.00 | .083 |
| **Target x Label (self)** | **0.214 [0.155, 0.273]** | **7.09** | **934.36** | **< .001** |
| Target x Label (social) | -0.019 [-0.059, 0.022] | 0.91 | 922.01 | .363 |
| **Domain x Label (self)** | **0.126 [0.061, 0.191]** | **3.78** | **559.80** | **< .001** |
| **Domain x Label (social)** | **0.144 [0.099, 0.189]** | **6.34** | **553.71** | **< .001** |
| Target x Puberty^2^ | -0.002 [-0.010, 0.006] | 0.54 | 250.61 | .590 |
| Domain x Puberty^2^ | -0.009 [-0.019, 0.000] | 1.90 | 488.05 | .058 |
| Label (self) x Puberty^2^ | -0.012 [-0.029, 0.004] | 1.51 | 364.83 | .133 |
| Label (social) x Puberty^2^ | -0.001 [-0.012, 0.010] | 0.18 | 361.86 | .859 |
| Puberty x Target x Domain | -0.014 [-0.037, 0.008] | 1.25 | 59.99 | .217 |
| Puberty x Target x Label (self) | 0.027 [-0.002, 0.055] | 1.81 | 42548.18 | .070 |
| Puberty x Target x Label (social) | 0.008 [-0.011, 0.028] | 0.84 | 42393.87 | .403 |
| Puberty x Domain x Label (self) | 0.021 [-0.008, 0.051] | 1.42 | 2436.32 | .156 |
| Puberty x Domain x Label (social) | 0.007 [-0.013, 0.027] | 0.68 | 2425.23 | .498 |
| **Target x Domain x Label (self)** | **-0.129 [-0.240, -0.017]** | **2.27** | **10051.42** | **.023** |
| Target x Domain x Label (social) | 0.055 [-0.021, 0.131] | 1.43 | 9909.85 | .154 |
| Target x Domain x Puberty^2^ | 0.002 [-0.014, 0.018] | 0.22 | 254.57 | .825 |
| Target x Label (self) x Puberty^2^ | -0.022 [-0.048, 0.005] | 1.60 | 3117.60 | .109 |
| Target x Label (social) x Puberty^2^ | 0.002 [-0.016, 0.020] | 0.18 | 3079.81 | .857 |
| Domain x Label (self) x Puberty^2^ | -0.003 [-0.029, 0.024] | 0.20 | 2283.92 | .843 |
| Domain x Label (social) x Puberty^2^ | 0.005 [-0.013, 0.023] | 0.50 | 2256.54 | .615 |
| Puberty x Target x Domain x Label (self) | -0.041 [-0.099, 0.016] | 1.41 | 11161.69 | .160 |
| Puberty x Target x Domain x Label (social) | -0.025 [-0.064, 0.014] | 1.25 | 11109.72 | .212 |
| **Target x Domain x Label (self) x Puberty^2^** | **0.101 [0.049, 0.153]** | **3.82** | **8145.71** | **< .001** |
| Target x Domain x Label (social) x Puberty^2^ | 0.020 [-0.015, 0.055] | 1.11 | 8046.81 | .268 |
|  |  |  |  |  |
| Random effects | *SD* |  |  |  |
| Parcellation |  |  |  |  |
| Intercept | 0.438 |  |  |  |
| Puberty | 0.044 |  |  |  |
| Puberty^2^ | 0.021 |  |  |  |
| Target | 0.046 |  |  |  |
| Domain | 0.076 |  |  |  |
| Puberty x Target | 0.003 |  |  |  |
| Puberty^2^ x Target | 0.013 |  |  |  |
| Puberty x Domain | 0.017 |  |  |  |
| Puberty^2^ x Domain | 0.014 |  |  |  |
| Target x Domain | 0.029 |  |  |  |
| Puberty x Target x Domain | 0.014 |  |  |  |
| Puberty^2^ x Target x Domain | 0.015 |  |  |  |
| Participant |  |  |  |  |
| Intercept | 0.045 |  |  |  |
| Puberty | 0.028 |  |  |  |
| Target | 0.033 |  |  |  |
| Domain | 0.068 |  |  |  |
| Puberty x Target | 0.025 |  |  |  |
| Puberty x Domain | 0.057 |  |  |  |
| Target x Domain | 0.085 |  |  |  |
| Puberty x Target x Domain | 0.056 |  |  |  |
| Residual | 0.816 |  |  |  |
| *Note.* Degrees of freedom (*df*) were calculated using the Satterthwaite approximation. | | | | |

**Discussion.** These results are largely in line with the results reported in the main text using age as a maturational index. However, there are two notable differences. First, collapsed across self and other evaluation, there was a positive rather than negative developmental trajectory in salience for social relative to academic information in social brain regions, although it was not statistically significant. Second, in self-related brain regions, we observed a U-shaped rather than an inverted U-shaped developmental trajectory for the self relative to other evaluation about social information. This difference appears to be driven by increased sensitivity to social information about others during mid-adolescence in the pubertal model, whereas it was associated with decreased sensitivity during this period in the chronological age model.

**Self-reported and behavioral analyses**

Here, we present supplementary analyses examining social and academic self-development across adolescence. At each wave, participants completed the self/other evaluation task, which indexes subjective social status and verbal academic competence, while in the MRI scanner. In addition, they also completed the Self-Perception Profile for Children (SPPC; Harter, 1985) and reported their social and academic competence at each wave, and the importance of social status and academic competence at waves 2 and 3.

We used multilevel modeling and model comparison to characterize the developmental trajectories of self-reported competence and importance from the SPPC, and subjective social status and verbal academic competence from the self/other evaluation task. Models were implemented in R 3.6.3. (R Core Team, 2018; https://www.r-project.org/) using the lmer function from the lme4 package (Version 1.1-25; Bates et al., 2015). Across all models, random effects were determined by selecting the least constrained set of parameters that converged in all model specifications within a comparison set (Barr et al., 2014). Maturation variables used in the analyses described below are age and pubertal development. We centered the age variable at 13 years and pubertal stage was centered at 3. Models were compared using the Akaike Information Criterion (AIC). A decrease in AIC of at least 2 points was considered to be a better fitting. For equivalently fitting models, the more parsimonious model was selected for interpretation.

**Self/other evaluation task analyses**

**Data cleaning.** To recover responses which occurred outside the 3s trial window or trials in which multiple responses were made (*n_wave1_* = 6.5%, *n_wave2_* = 5.0%, *n_wave3_* = 2.8%), we developed a recoding scheme based on the distribution of response times. We recoded responses based on the following rules: 1) if the response for a given trial was missing and the reaction time of the next trial was < 1000ms and had multiple response, we recoded the trial as the first response and the next trial as the next response; 2) if the response for a given trial was missing and the reaction time of the next trial was < 750ms and had a single response, we recoded the trial as the response and the next trial as missing; 3) if a trial had multiple responses, we recoded the trial as the last response. All code related to the development and implementation of the recoding criteria is available online (https://github.com/dsnlab/social_reorientation/blob/main/analysis/clean_behavioral_data/task.Rmd).

**Multilevel modeling procedure.** We specified a base model and four polynomial growth models to characterize the best fitting developmental trajectory of subjective social status and verbal academic competence measured in the self/other evaluation task with either age or puberty as the maturation index. Criterion variables for subjective social status and verbal academic competence were created by determining the percentage of completed trials in which participants endorsed positive items and rejected negative items. Higher scores in the social domain indicate higher subjective social status, whereas higher scores in the academic domain indicate greater subjective verbal academic competence. In the base model, criterion variables at each wave were regressed on the fixed effects of the intercept, Domain, Target, and the interaction between Domain and Target. In the growth models, the following terms were added: the linear effect of Maturation (Model 1), the linear and quadratic effects of Maturation (Model 2), the linear effect of Maturation, its interactions with Domain and Target, and the three-way interaction between these variables (Model 3), and the linear and quadratic effects of Maturation and their respective two- and three-way interactions with Domain and Target (Model 4). In all models, the intercept, Domain, Target, and the interaction between Domain and Target were allowed to vary across participants as random effects. We compared model fit for each maturation index separately.

**Results.** We assessed developmental effects of subjective social status and verbal academic competence reported during the self/other evaluation task by comparing multilevel polynomial growth models. For chronological age, Model 4 best fit the data, whereas for puberty, Model 3 best fit the data (Table S5).

| Table S5  *Comparison of Polynomial Growth Models Regressing Percent Endorsement During the Self/Other Evaluation Task on Predictors* | | |
| --- | --- | --- |
| Model | Model *df* | AIC |
| Base – Domain x Target | 15 | 5237.97 |
|  | Age | |
| 1 – Domain x Target + Age | 16 | 5239.91 |
| 2 – Domain x Target + Age + Age^2^ | 17 | 5241.69 |
| 3 – Domain x Target x Age | 19 | 5233.46 |
| **4 – Domain x Target x Age + Domain x Target x Age^2^** | **23** | **5231.07** |
|  | Puberty | |
| 1 – Domain x Target + Puberty | 16 | 5238.96 |
| 2 – Domain x Target + Puberty + Puberty^2^ | 17 | 5238.77 |
| **3 – Domain x Target x Puberty** | **19** | **5232.81** |
| 4 – Domain x Target x Puberty + Domain x Target x Puberty^2^ | 23 | 5237.02 |
| *Note*. The best fitting models are bolded. An AIC difference of at least 2 points was used as the criterion for a better fitting model. | | |

In both models, there were interactions between Domain and Target, such that the difference between one’s perceived social status and verbal academic competence was greater when evaluating oneself compared to evaluating a fictional other (Figure S9). There was also a 3-way interaction with the linear effect of maturation, such that one’s own subjective social status increased linearly across adolescence, but these effect was not statistically significant in either the age (*b* = 2.09, 95% CI [-0.04, 4.22], *p* = .056) or pubertal model (*b* = 3.53, 95% CI [-0.78, 7.83], *p* = .109).


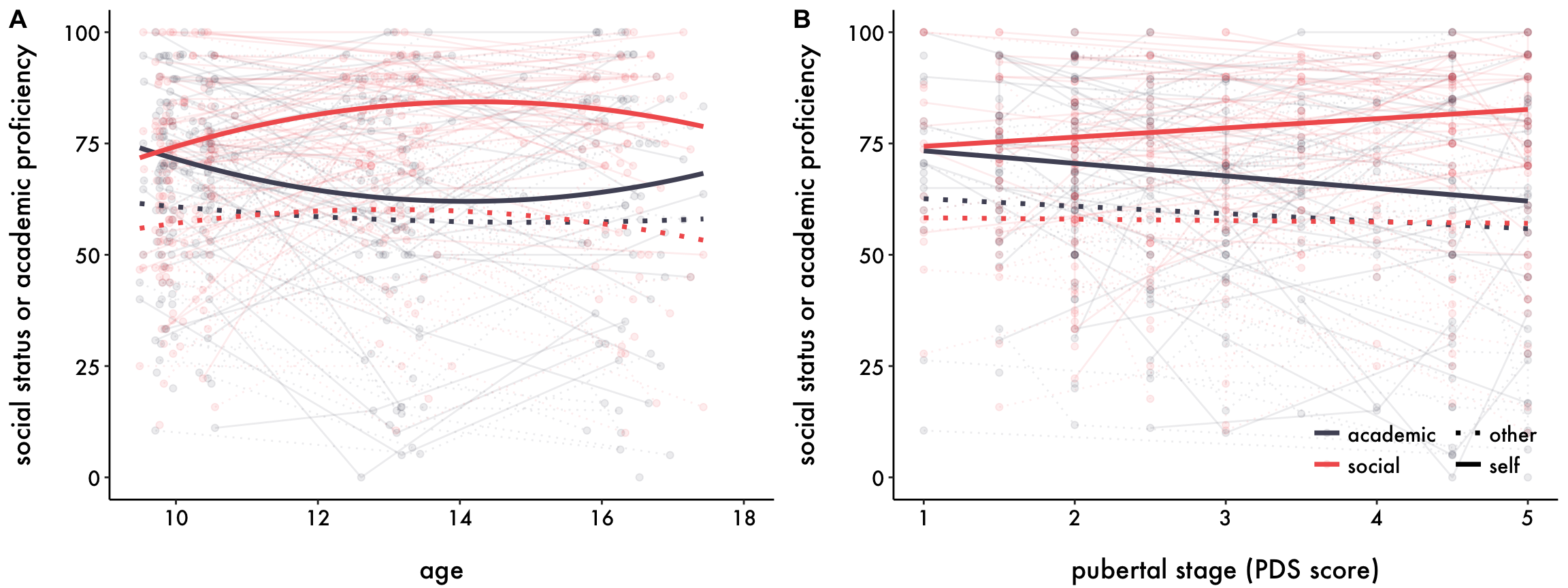


*Figure S9.* Developmental trajectories for subjective social status and verbal academic competence as a function of Domain and Target for A) chronological age, and B) puberty. Thin lines and points represent individual data points and change between waves, whereas thick lines represent fitted average developmental trajectories from best fitting models.

| Table S6  *Results of the Best Fitting Multilevel Models with Percent Endorsement During the Self/Other Evaluation Task as the Criterion* | | | | | |
| --- | --- | --- | --- | --- | --- |
|  | Age | | | | |
| Fixed effects | *b* [95% CI] | *SE* | *t* | *df* | *p* |
| **Intercept (academic, other, age 13)** | **57.87 [51.28, 64.47]** | **3.36** | **17.21** | **174.40** | **< .001** |
| Target | 4.82 [-4.34, 13.97] | 4.67 | 1.03 | 182.28 | .304 |
| Domain | 2.29 [-6.42, 11.01] | 4.45 | 0.52 | 188.62 | .607 |
| Age | -0.56 [-1.65, 0.53] | 0.55 | 1.01 | 365.43 | .312 |
| Age^2^ | 0.14 [-0.42, 0.69] | 0.28 | 0.48 | 347.30 | .631 |
| **Target x Domain** | **18.51 [6.41, 30.61]** | **6.17** | **3.00** | **194.63** | **.003** |
| Target x Age | -0.68 [-2.22, 0.86] | 0.79 | 0.87 | 366.83 | .386 |
| Domain x Age | 0.55 [-0.97, 2.07] | 0.78 | 0.70 | 371.77 | .481 |
| Target x Age^2^ | 0.43 [-0.35, 1.21] | 0.40 | 1.07 | 348.69 | .283 |
| Domain x Age^2^ | -0.48 [-1.26, 0.29] | 0.40 | 1.22 | 352.91 | .222 |
| Target x Domain x Age | 2.09 [-0.04, 4.22] | 1.09 | 1.92 | 372.86 | .056 |
| Target x Domain x Age^2^ | -0.64 [-1.73, 0.46] | 0.56 | 1.14 | 356.85 | .255 |
|  |  |  |  |  |  |
| Random effects | variance | *SD* |  |  |  |
| Participant |  |  |  |  |  |
| Intercept | 377.05 | 19.42 |  |  |  |
| Target | 689.50 | 26.26 |  |  |  |
| Domain | 565.08 | 23.77 |  |  |  |
| Target x Domain | 1040.78 | 32.26 |  |  |  |
| Residual | 239.13 | 15.46 |  |  |  |
|  |  | | | | |
|  | Puberty | | | | |
| Fixed effects | *b* [95% CI] | *SE* | *t* | *df* | *p* |
| **Intercept (academic, other, stage 3)** | **59.23 [54.19, 64.28]** | **2.57** | **23.01** | **78.80** | **< .001** |
| **Target** | **8.48 [1.51, 15.45]** | **3.56** | **2.38** | **80.81** | **.020** |
| Domain | -1.54 [-8.03, 4.94] | 3.31 | 0.47 | 80.29 | .642 |
| Puberty | -1.68 [-3.91, 0.54] | 1.13 | 1.48 | 380.92 | .139 |
| **Target x Domain** | **12.33 [3.43, 21.24]** | **4.54** | **2.71** | **80.80** | **.008** |
| Target x Puberty | -1.14 [-4.28, 2.00] | 1.60 | 0.71 | 379.97 | .478 |
| Domain x Puberty | 1.37 [-1.72, 4.46] | 1.57 | 0.87 | 382.90 | .385 |
| Target x Domain x Puberty | 3.53 [-0.78, 7.83] | 2.20 | 1.60 | 383.96 | .109 |
|  |  |  |  |  |  |
| Random effects | variance | *SD* |  |  |  |
| Participant |  |  |  |  |  |
| Intercept | 367.36 | 19.17 |  |  |  |
| Target | 687.68 | 26.22 |  |  |  |
| Domain | 561.36 | 23.69 |  |  |  |
| Target x Domain | 1030.63 | 32.10 |  |  |  |
| Residual | 244.39 | 15.63 |  |  |  |
| *Note.* Degrees of freedom (*df*) were calculated using the Satterthwaite approximation. Statistically significant parameters at *p* < .05 are bolded. Pubertal stage was centered at 3; age was centered at 13 years. The reference group for Domain was Academic; the reference group for Target was Other. | | | | | |

**Self-reported competence and importance analyses**

**Self-report measures.** At all waves, participants rated their social and academic competence via the Self-Perception Profile for Children (SPPC; Harter, 1985). Within each domain, participants rated 6 items using a 4-point scale. For *competence*, items in the academic domain measure general scholastic competence (e.g., doing well in school, easily completing homework), while items in the social domain measure social skill competence (e.g., knowing how to make friends and become popular). Participants also rated 6 items measuring global self-worth, or self-esteem. At waves 2 and 3, participants also rated the *importance* of academic achievement and social status for self-esteem, each measured with 2 items. Items are formatted in a structured alternative format devised to reduce social desirability; participants first determine which kind of children they are like (e.g., “Some kids find it hard to make friends” or “Other kids find it’s pretty easy to make friends”) and then rate whether the selected statement is “really true” or “sort of true” for them. In this sample, social and academic competence (*r*  = .31, 95% CI [.12, .48], *p* = .002) and importance for self-worth (*r*  = .12, 95% CI [-.23, .43], *p* = .495) are weakly correlated within-person.

**Multilevel modeling procedure.** We used multilevel modeling and model comparison to characterize the developmental trajectories of self-reported competence ,importance, and global self-worth from the SPPC. The same modeling procedure for the self/other evaluation task was used for the competence models. However, because importance was only measured at the second and third waves, only the linear fixed effects of Maturation can be interpreted. Therefore, we compared a base model to just two linear growth curve models for each maturation index. In all importance models mean importance ratings at each wave were regressed on the fixed effects of the intercept and Domain, and the intercept was treated as a random effect across participants. In the growth curve models, we added the linear fixed effects of Maturation (Model 1), and the interaction between this effect and Domain (Model 2). For the models assessing developmental changes in global self-worth, ratings were regressed on the linear and/or quadratic effects of Maturation, and intercepts and linear slopes were allowed to vary randomly across participants.

| Table S7  *Comparison of Polynomial Growth Models of Competence Ratings* | | |
| --- | --- | --- |
| Model | Model *df* | AIC |
| Base – Domain | 4 | 491.88 |
|  | Age | |
| 1 – Domain + Age | 7 | 490.33 |
| **2 – Domain + Age + Age^2^** | **8** | **488.18** |
| 3 – Domain x Age | 8 | 492.32 |
| 4 – Domain x Age + Domain x Age^2^ | 10 | 492.15 |
|  | Puberty | |
| 1 – Domain + Puberty | 7 | 493.55 |
| 2 – Domain + Puberty + Puberty^2^ | 8 | 494.34 |
| 3 – Domain x Puberty | 8 | 495.54 |
| 4 – Domain x Puberty + Domain x Puberty^2^ | 10 | 497.65 |
| *Note*. The best fitting models are bolded. An AIC difference of at least 2 points was used as the criterion for a better fitting model. | | |

**Competence results.** For age, model comparison revealed that Model 2 best fit the data, whereas no puberty model fit the data better than the base model (Table S7). As such, we will interpret and visualize only the age model. In this model, we observed a statistically significant fixed effect of Domain, such that competence ratings in the social domain were lower than in the academic domain; this effect did not change as a function of age. We observed a small positive linear effect of age and a small negative quadratic effect of age, though the linear effect was not statistically different from zero. All statistics are provided in Table S8 and visualized in Figure S10A.


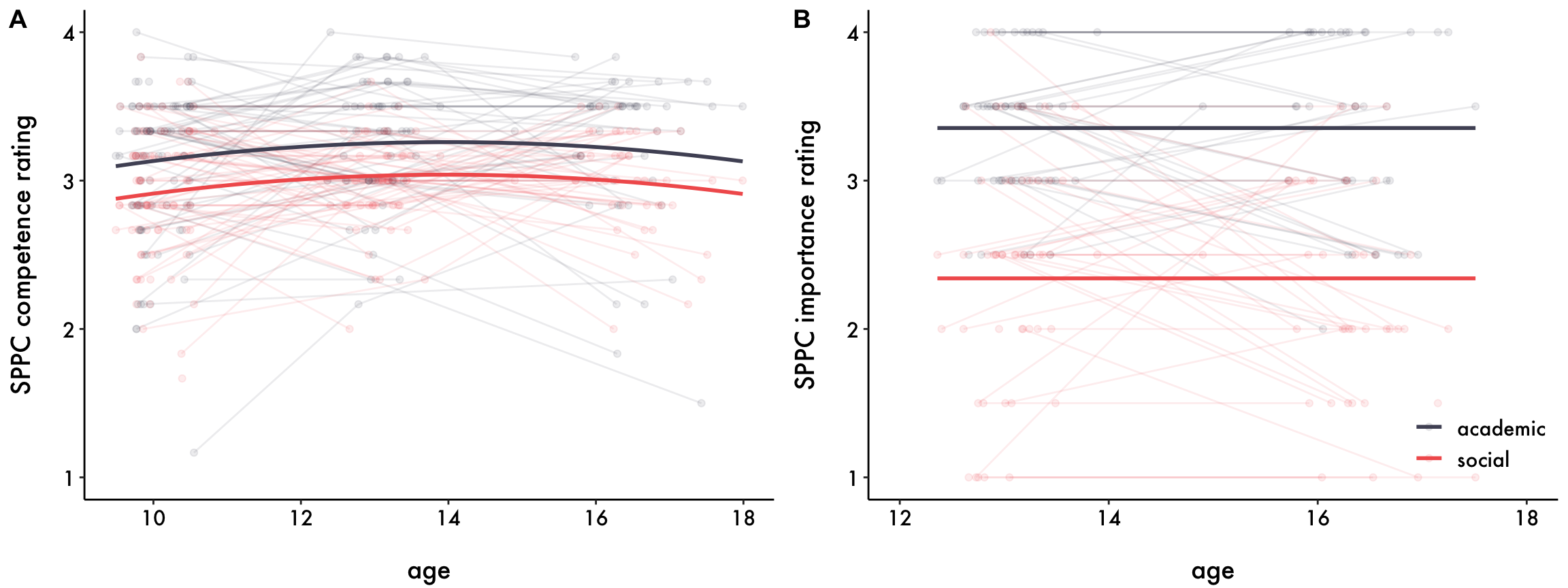


*Figure S10.* Developmental trajectories of self-reported social and academic A) competence and B) importance for self-esteem reported in the SPPC. Thin lines and points represent individual data points and change between waves, whereas thick lines represent fitted average developmental trajectories from best fitting models.

| Table S8  *Results of the Best Fitting Multilevel Models of Competence, Importance, and Global Self-worth Ratings* | | | | | |
| --- | --- | --- | --- | --- | --- |
|  | Competence | | | | |
| Fixed effects | *b* [95% CI] | *SE* | *t* | *df* | *p* |
| **Intercept (academic, age 13)** | **3.26 [3.16, 3.36]** | **0.05** | **62.97** | **227.38** | **< .001** |
| **Domain (social)** | **-0.13 [-0.22, -0.05]** | **0.04** | **2.99** | **223.05** | **.003** |
| Age | 0.02 [0.00, 0.04] | 0.01 | 1.96 | 65.85 | .054 |
| Age^2^ | **-0.01 [-0.02, -0.00]** | **0.01** | **2.03** | **317.50** | **.043** |
|  |  |  |  |  |  |
| Random effects | variance | *SD* |  |  |  |
| Participant |  |  |  |  |  |
| Intercept | 0.04 | 0.20 |  |  |  |
| Age | 0.00 | 0.04 |  |  |  |
| Residual | 0.18 | 0.42 |  |  |  |
|  |  |  |  |  |  |
|  | Importance | | | | |
| Fixed effects | *b* [95% CI] | *SE* | *t* | *df* | *p* |
| **Intercept (academic, age 13)** | **3.35 [3.23, 3.48]** | **0.06** | **52.25** | **55.06** | **< .001** |
| **Domain (social)** | **-1.01 [-1.21, -0.81]** | **0.10** | **10.00** | **55.63** | **< .001** |
|  |  |  |  |  |  |
| Random effects | variance | *SD* |  |  |  |
| Participant |  |  |  |  |  |
| Intercept | 0.05 | 0.23 |  |  |  |
| Residual | 0.36 | 0.60 |  |  |  |
|  |  | | | | |
|  | Global Self-worth | | | | |
| Fixed effects | *b* [95% CI] | *SE* | *t* | *df* | *p* |
| **Intercept (stage 3)** | **3.23 [3.15, 3.31]** | **0.04** | **78.35** | **85.14** | **< .001** |
| Puberty | -0.04 [-0.09, 0.00] | 0.02 | 1.93 | 62.45 | .058 |
|  |  |  |  |  |  |
| Random effects | variance | *SD* |  |  |  |
| Participant |  |  |  |  |  |
| Intercept | 0.09 | 0.30 |  |  |  |
| Puberty | 0.01 | 0.09 |  |  |  |
| Residual | 0.10 | 0.31 |  |  |  |
| Note. Degrees of freedom (df) were calculated using the Satterthwaite approximation. Statistically significant parameters at *p* < .05 are bolded. Age was centered at 13 years; puberty was centered at stage 3). The reference group for Domain was Academic. | | | | | |

**Importance results.** We investigated developmental changes in the importance of social status and academics for self-esteem across waves 2 and 3 by fitting a series of multilevel models and comparing them. Overall, the base model without growth parameters was the best fitting (Table S9). In this model, we observed a statistically significant fixed effect of Domain, such that social status was rated as being less important for self-esteem than academics (Figure S10B; Table S8).

| Table S9  *Comparison of Polynomial Growth Models of Importance Ratings* | | |
| --- | --- | --- |
| Model | Model *df* | AIC |
| **Base – Domain** | **6** | **356.28** |
| 1 – Domain + Age | 7 | 356.65 |
| 1 – Domain + Puberty | 7 | 357.77 |
| 2 – Domain + Age + Age^2^ | 8 | 356.69 |
| 2 – Domain + Puberty + Puberty^2^ | 8 | 359.27 |
| *Note*. The best fitting model is bolded. An AIC difference of at least 2 points was used as the criterion for a better fitting model. | | |

**Global self-worth results.** We investigated developmental changes in global self-worth by fitting a series of multilevel models and comparing them. Overall, only the model including the linear effect of puberty fit the data better than the base model (Table S10). In this model, pubertal stage was associated with lower global self-worth, but this relationship was not statistically different from zero (Figure S11; Table S8).

| Table S10  *Comparison of Polynomial Growth Models of Global Self-worth Ratings* | | |
| --- | --- | --- |
| Model | Model *df* | AIC |
| Base – Intercept only | 3 | 210.90 |
| 1 – Age | 6 | 209.63 |
| **1 – Puberty** | **6** | **208.18** |
| 2 – Age + Age^2^ | 7 | 209.33 |
| 2 – Puberty + Puberty^2^ | 7 | 209.56 |
| *Note*. The best fitting model is bolded. An AIC difference of at least 2 points was used as the criterion for a better fitting model. | | |


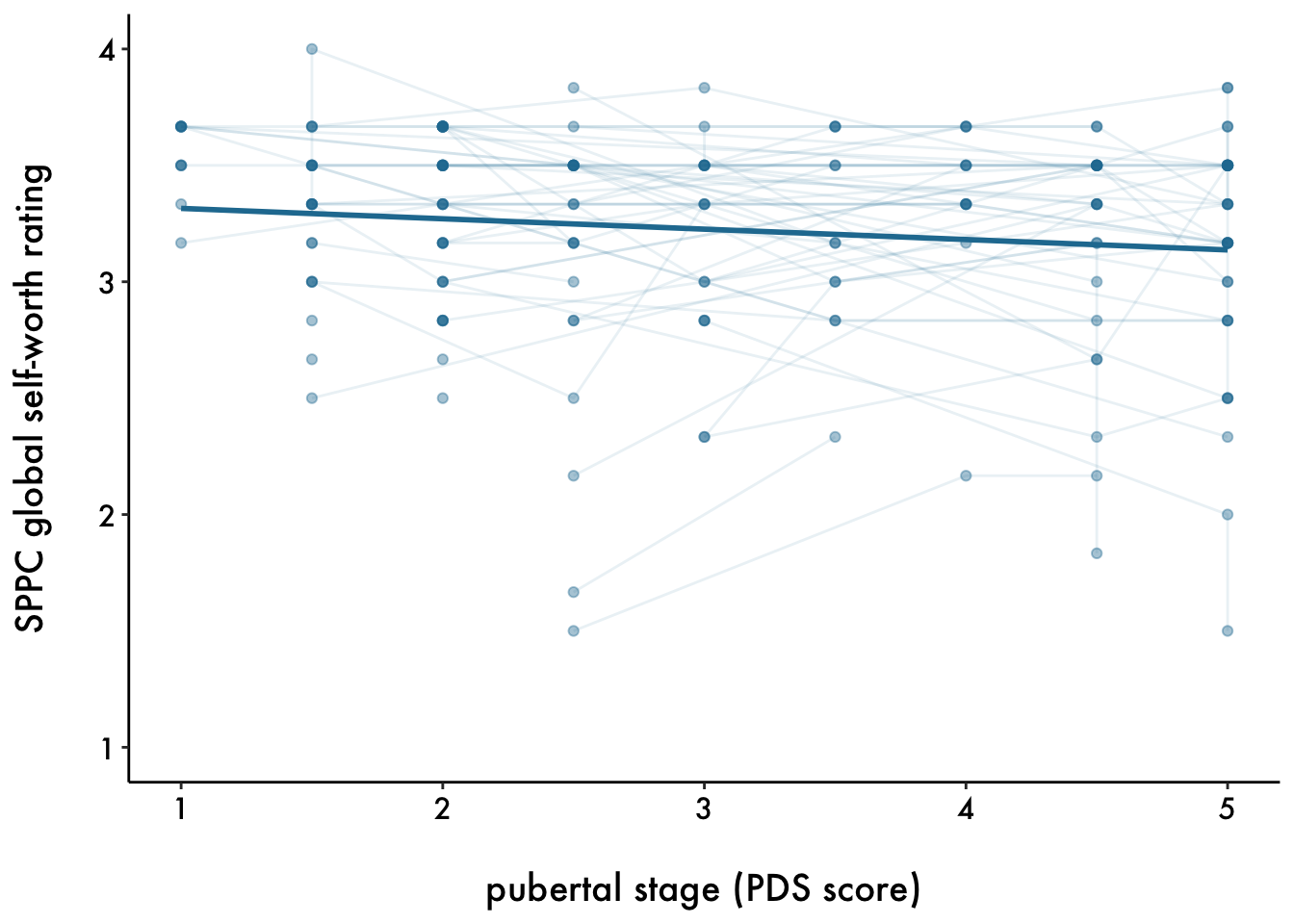


*Figure S11.* Developmental trajectories of self-reported global self-worth reported in the SPPC. Thin lines and points represent individual data points and change between waves, whereas thick lines represent fitted average developmental trajectories from the best fitting model.
